# Subsidiary closures and relocations in the multinational enterprise: Reinstating cooperation in subsidiaries to enable knowledge transfer

**DOI:** 10.1057/s41267-022-00592-w

**Published:** 2023-01-31

**Authors:** Marty Reilly, Esther Tippmann, Pamela Sharkey Scott

**Affiliations:** 1 DCU Business School, Dublin City University, Dublin, Ireland; 2 University of Galway, J. E. Cairnes School of Business and Economics, Galway, Ireland

**Keywords:** knowledge transfer, identity, subsidiary relocation, subsidiary closure/divestment, MNE/MNC, emotions

## Abstract

Subsidiary closures and relocations, a process whereby a multinational enterprise (MNE) closes down a subsidiary and relocates its activities, are commonplace and increasing. Yet we lack an understanding of how knowledge in such situations can be successfully transferred to prevent loss and provide for future knowledge recombination in the MNE. Compared to periods of normal operation, knowledge sharing during subsidiary relocations is likely compromised by diminished sender motivation. In a detailed case study of a subsidiary closure and relocation, we find that the announcement of a subsidiary closure can lead to a break in cooperative behavior that inhibits knowledge transfer. It is therefore critical to reinstate cooperative behavior among subsidiary employees. Reinstatement can be achieved through a set of subsidiary leadership practices that affect the emotions of employees and subsidiary identity. This finding contributes to our understanding of knowledge transfer dynamics in MNEs during subsidiary relocations and closures, extends theory on the practices of subsidiary leadership in subsidiary death and adds to our understanding of identity in MNEs.

## Résumé

Les fermetures et délocalisations de filiales, processus par lequel une entreprise multinationale (*Multinational Enterprise* – MNE) ferme une filiale et délocalise ses activités, sont fréquentes et ne cessent d’augmenter. Pourtant, nous ne comprenons pas comment, dans de telles situations, les connaissances peuvent être transférées avec succès pour en éviter la perte et permettre une future recombinaison des connaissances dans la MNE. Par rapport aux périodes de fonctionnement normal, le partage des connaissances pendant les délocalisations de filiales est probablement compromis par la diminution de la motivation de l'expéditeur. Dans une étude de cas détaillée sur la fermeture et la délocalisation d'une filiale, nous observons que l'annonce de la fermeture d'une filiale peut entraîner une rupture du comportement coopératif qui inhibe le transfert de connaissances. Il est donc essentiel de rétablir un comportement coopératif parmi les employés de la filiale. Ce rétablissement peut se faire par le biais d'un ensemble de pratiques de leadership de la filiale qui influence les émotions des employés et l'identité de cette dernière. Ce résultat contribue à notre compréhension de la dynamique du transfert de connaissances dans les MNEs lors des délocalisations et des fermetures de filiales. Il élargit également la théorie des pratiques de leadership de la filiale dans la mort de celle-ci et enrichit notre compréhension de l'identité dans les MNEs.

## Resumen

Los cierres y reubicación de las filiales, un proceso mediante el cual una empresa multinacional (EMN) cierra una filial y reubica sus actividades, son habituales y están aumentando. Empero, nos falta un entendimiento de cómo el conocimiento en dadas situaciones puede ser transferido de manera exitosa para prevenir perdidas y contempla recombinación de conocimiento futuro en las empresas multinacionales. En comparaciones de operación normal, compartir conocimiento durante la reubicación de una filial podría verse afectada por una disminución de la motivación del emisor. En un estudio de caso detallado de un cierre de una filial y la reubicación, encontramos que el anuncio del cierre de una filial puede llevar a una interrupción del comportamiento cooperativo e inhibir la transferencia de conocimiento. Por esto es crítico restaurar el comportamiento cooperativo entre los empleados. La restauración puede ser consigo mediante un conjunto de prácticas de liderazgo que afectan las emociones de los empleados y la identidad de las filiales. Estos hallazgos contribuyen a nuestro entendimiento de las dinámicas de transferencia de conocimiento en las empresas multinacionales durante la reubicación y cierre de las filiales, avanza la teoría y la práctica de liderazgo en el deceso de las filiales y añade a nuestro entendimiento de la identidad en las empresas multinacionales.

## Resumo

Fechamentos e realocações de subsidiárias, um processo pelo qual uma empresa multinacional (MNE) fecha uma subsidiária e realoca suas atividades, são comuns e estão aumentando. No entanto, carecemos de um entendimento de como conhecimento em tais situações pode ser transferido com sucesso para evitar perdas e propiciar uma futura recombinação de conhecimento na MNE. Em comparação com períodos de normal operação, compartilhamento de conhecimento durante realocações de subsidiárias provavelmente é comprometido pela reduzida motivação do emitente. Em um estudo de caso detalhado do fechamento e realocação de uma subsidiária, descobrimos que o anúncio do fechamento de uma subsidiária pode levar a uma quebra do comportamento cooperativo que inibe a transferência de conhecimento. Portanto, é fundamental restabelecer o comportamento cooperativo entre funcionários da subsidiária. A reintegração pode ser alcançada por meio de um conjunto de práticas de liderança da subsidiária que afetam as emoções dos funcionários e a identidade da subsidiária. Essa descoberta contribui para nossa compreensão da dinâmica de transferência de conhecimento em MNEs durante relocações e fechamentos de subsidiárias, amplia a teoria sobre as práticas de liderança da subsidiária na extinção da subsidiária e aumenta nossa compreensão sobre identidade em MNEs.

## Abstract (zh)

子公司关闭和搬迁是跨国企业 (MNE) 关闭子公司并重新安置其活动的过程， 这种情况司空见惯， 而且越来越多。然而， 我们不了解在这种情况下知识如何能够成功转移以防止损失并为MNE的未来知识重组做准备。与正常运营时期相比， 子公司搬迁期间的知识共享可能会因减弱的发送者动机而受到损害。在对子公司关闭和搬迁的一个详细案例研究中， 我们发现宣布关闭子公司会导致合作行为中断， 从而抑制知识转移。因此， 恢复子公司员工之间的合作行为至关重要。恢复可以通过一系列影响员工情绪和子公司身份的子公司领导力实践来实现。这一发现有助于我们了解MNE在子公司搬迁和关闭期间的知识转移动态， 扩展在子公司消亡中子公司领导力实践的理论， 并增加我们对MNE身份的理解。

## INTRODUCTION

Subsidiary closures and relocations – and their associated knowledge transfers – are a common and increasingly frequent aspect of managing the multinational enterprise (MNE) (Belderbos & Zou, 2006; UNCTAD, 2020). As MNEs continuously manage their international footprint, closure and relocation may represent the natural, final step in a subsidiary’s evolution (e.g., Birkinshaw, 1996; Birkinshaw & Hood, 1998). Subsidiary closure and relocations are also increasingly frequent due to a range of factors, including trends for shorter and less fragmented global value chains; re-shoring and near-shoring of value chain activities (Kano, Tsang, & Yeung, 2020); the rise of more populist policies urging MNEs to bring foreign operations back home (Hartwell & Devinney, 2021; Wu, Strange, & Shirodkar, 2021); international sanctions, invasions, and wars that require withdrawal from certain countries (Sonnenfeld et al., 2022); and demands for flexibility in MNE location strategies to capitalize on changing opportunities for value creation in cross-border activities (Berry, 2010; Konara & Ganotakis, 2020; Song, 2014). Indeed, the longer-term implications of the COVID-19 pandemic are expected to include an increase in subsidiary closures and relocations (UNCTAD, 2020).

Subsidiary closures and relocations involve the movement of subsidiary activities to another internal unit within the MNE’s portfolio of affiliates or partner organizations before the subsidiary is closed (Belderbos & Zou, 2009; Nachum & Song, 2011; Schmid & Morschett, 2020). A relocation therefore requires knowledge transfers from the closing entity to other units. In the aftermath of a subsidiary closure announcement, there is a wind-down process to transfer business-critical mandates and knowledge (both tacit and organizationally complex knowledge) to safeguard business continuity and enable future knowledge recombination for the MNE. As effective knowledge transfer processes are critical for the MNE during a subsidiary relocation, this paper addresses knowledge outflows from a closing subsidiary as the “sender”.

There is a vast body of insights into knowledge transfers in MNEs (e.g., Gupta & Govindarajan, 1991, 2000; Noorderhaven & Harzing, 2009; Zeng, Grøgaard, & Steel, 2018). This includes knowledge outflows from subsidiaries to other MNE units (e.g., Liu & Meyer, 2020; Monteiro, Arvidsson, & Birkinshaw, 2008; Mudambi, Piscitello, & Rabbiosi, 2014). A particularly relevant insight is the finding that motivation is key for subsidiaries to share their knowledge (Minbaeva, 2007; Minbaeva & Michailova, 2004), as knowledge transfer requires both willingness and commitment from the sender. But during subsidiary closures and relocations, we cannot assume that subsidiary employees will continue to be motivated to share knowledge. One reason is that employees will need to find new employment. There is thus a risk that an employee with important tacit knowledge will depart before this knowledge can be transferred. Another reason is that even if most subsidiary employees stay on (either until the eventual closure of the subsidiary or by moving to another MNE unit), they will likely experience emotional responses of anger and frustration – typical responses following a closure announcement for an organization (Crosina & Pratt, 2019; Sutton, 1987). Such responses can undermine their motivation to transfer knowledge to other units.

However, prior studies of knowledge transfers from subsidiaries focus on the sharing of processes and practices by the sending unit during normal and continuing operations (e.g., Noorderhaven & Harzing, 2009; Zeng et al., 2018). As these studies are contextualized in situations where the sending subsidiary continues to exist, they do not address how knowledge transfer occurs in a situation where the subsidiary is destined to close and sender motivation may be compromised. Given the need for an MNE to enable the transfer of business-critical knowledge to other units ahead of subsidiary closure to avoid knowledge loss, insights into knowledge transfers during subsidiary relocation are important. We therefore ask: how is knowledge transferred during a subsidiary closure and relocation?

This paper presents a longitudinal case study of a subsidiary, Gamma, as it was relocated. We gathered detailed qualitative data on what happened between the announcement of the decision and subsidiary closure one year later. The case is revelatory because it involved the relocation of all of Gamma’s activities to other internal units and external organizations. In other words, none of the subsidiary activities was terminated. This made knowledge transfer a very salient aspect including the transfer of business-critical, tacit, and organizationally complex knowledge. Moreover, similar to situations of organizational death (Harris & Sutton, 1986; Sutton, 1987), the relocation of activities was experienced by employees as the subsidiary “dying” due to the permanent closure of the unit, the displacement of subsidiary employees, and an associated sense of loss akin to the loss of a relative or friend. We therefore refer to the subsidiary closure and relocation of activities as “subsidiary death”.^1^

We found that the closure announcement disrupted the subsidiary’s established identity within the MNE, and evoked emotions of anger and distrust among employees. This instigated emotional barriers that initially rendered the transfer of knowledge impossible. The emergence of a legacy subsidiary identity was critical to enabling knowledge transfer. The emergence of such a legacy identity within the subsidiary was facilitated by a set of leadership practices exercised by subsidiary managers (rather than headquarter managers), which served to reinstate cooperative behavior for knowledge sharing by employees. These leadership practices included engaging with emotions, reconfiguring incentives, and sensegiving to support a legacy subsidiary identity. We summarize these findings in our model of knowledge transfer during subsidiary death.

The main theoretical implications are threefold. First, we advance theory on knowledge transfers in MNEs by exposing the dynamics of knowledge transfer during subsidiary closure and relocations. In particular, we explore how knowledge transfer can be enabled in situations when a subsidiary no longer shares the MNE’s identity. Our second contribution is to reveal the practices of subsidiary leadership in an often-overlooked but increasingly important part of subsidiary evolution, subsidiary death. Third, we add to the understanding of organizational identity within the MNE.

## THEORETICAL FRAMING

The capacity to share, integrate, and recombine knowledge across borders is a main source of advantage for MNEs (Doz, Santos, & Williamson, 2001; Kogut & Zander, 1993). The study of intra-organizational knowledge transfers has thus been central to international business scholarship. While we are not aware of a prior study that has investigated knowledge transfers during a subsidiary closure and relocation, the vast body of research into knowledge transfers in MNEs during steady states of continuing subsidiary operations offers some important insights on the mechanisms that may be at play. We detail these mechanisms below, elaborating also on the challenging conditions evoked in the context of subsidiary relocation.

### Knowledge Transfers in MNEs

In general, the characteristics of knowledge influence how it is transferred. As with most knowledge transfer situations, subsidiary relocations involve both codified and tacit knowledge of products, processes, and practices. In terms of the influence of knowledge characteristics on knowledge transfer, codified knowledge should transfer easily to other units (Kogut & Zander, 1996; Minbaeva, 2007). However, the sharing of tacit knowledge is challenging due to mobility barriers (Gupta & Govindarajan, 2000; Parker, Tippmann, & Kratochvil, 2019; Polanyi, 1966; Tallman & Chacar, 2011). For example, tacit knowledge sharing requires externalization by individuals through deep engagement with others (Nonaka, 1994; Nonaka & Takeuchi, 1995; Noorderhaven & Harzing, 2009). Moreover, organizational knowledge is complex, because it may be spread across various geographic locations or across multiple actors at different levels of the organization (Gaur, Ma, & Ge, 2019). Organizational knowledge is also often causally ambiguous (Szulanski, 1996; Tallman & Chacar, 2011). The ability to capture and share such knowledge therefore requires concerted and collective efforts by organizational members as well as significant interactions (Athanassiou & Nigh, 2000).

In addition to the characteristics of knowledge, knowledge transfers are influenced by the attributes of the sender and the receiver, their relationship, and the organizational context (Gao & Riley, 2010; Osterloh & Frey, 2000). In subsidiary relocations, the pressing issue is how the subsidiary to be closed engages in activities to transfer its knowledge to other units. Sender attributes are therefore key to understanding knowledge transfers. In terms of sender characteristics, the MNE knowledge transfer literature highlights the “disseminative capacity” of the sender as the ability and willingness of organizational members to share their knowledge (Minbaeva, 2007; Minbaeva & Michailova, 2004). This is because successful knowledge transfer rests on both the ability of the employees to communicate and share their knowledge as well as their willingness or motivation (Baldwin, 1959) to engage in the “distinct experience… of dissemination” (Szulanski, 1996: 28). Given the importance of employee motivation, the act of sharing knowledge with others can be described as “donating” knowledge (Lin, 2007: 136) or engagement in “voluntary work behavior” (Husted, Michailova, Minbaeva, & Pedersen, 2012: 754). Overall, many studies have investigated the impact of ability and willingness in various knowledge transfer contexts (Bakker, Leenders, Gabbay, Kratzer, & Van Engelen, 2006; Chang & Chuang, 2011; Cruz, Perez, & Cantero, 2009; Foss, Minbaeva, Pedersen, & Reinholt, 2009), concluding that both must be present for effective transfer (Minbaeva, Pederson, Bjorkman, Fey, & Park, 2003; Reinholt, Pedersen, & Foss, 2011).

Organizations must therefore find ways to ensure employee ability and willingness to transfer knowledge. Employee ability is supported by the organization’s human resource management practices of training, development, and performance appraisal (Minbaeva et al., 2003). But ensuring motivation is more complex, as it has both extrinsic and intrinsic dimensions. Extrinsic motivation for knowledge transfer is encouraged by linking rewards (e.g., financial bonuses, job security, or promotion) to achieving the goals of the unit and MNE (Calder & Staw, 1975; Osterloh & Frey, 2000). Intrinsic motivation has further subtleties, requiring employee identification with a shared MNE purpose and voluntary participation in acting consistent with this MNE identity (Osterloh & Frey, 2000). In this latter respect, it is important to note that the MNE is usually seen as a social community with a shared identity – or a meta-identity (Fortwengel, 2021) – that enables the speed and efficiency of knowledge sharing across units (Kogut & Zander, 1993, 1996). A MNE meta-identity reduces self-interested behavior, induces cooperation across units, and creates a common code for knowledge transfer (including behavioral norms that coordinate knowledge sharing and expect future reciprocity from the receiving unit) (Cabrera & Cabrera, 2002; Minbaeva, 2007). Further, employees can be motivated by identifying with the MNE because their “longing to belong” to this social community triggers behaviors desired by the corporation (Kogut & Zander, 1996: 502).

### Challenges During Subsidiary Closures and Relocations

So far, we have outlined the theoretical premises of knowledge transfers in relation to subsidiaries sharing knowledge under the assumption that their operations will continue to exist into the future. But for subsidiary closures and relocations, this is not the case, and for such situations the literature on organizational death in management studies emerged as very relevant to our study. This literature explores the collective process of organizational dying, ending with the actual death of the organization (or in our context, the death of the subsidiary). Following early work on organizational death, the metaphor of subsidiary death serves as a reminder that organizations are living and dying systems (Harris & Sutton, 1986), whereby death denotes the final stage of organizing (Van de Ven & Poole, 1995; Whetten, 1987). The metaphor also captures the sense of loss that people experience when an organization dies, which is similar to that of a friend or loved one dying (Crosina & Pratt, 2019; Harris & Sutton, 1986).

As in the case of organizational death (Sutton, 1987), the metaphor further includes the advance notice of subsidiary closure and an unambiguous end state of closure: there is no room for micro-political bargaining between a subsidiary and its headquarters to shape a future role (Dörrenbächer & Gammelgaard, 2011). Usually, subsidiary managers seek to exert influence on headquarters when repositioning mandates (Birkinshaw & Hood, 1998; Conroy, Collings, & Clancy, 2019; Tippmann, Sharkey Scott, Reilly, & O’Brien, 2018), including during MNE restructuring processes (Balogun, Fahy, & Vaara, 2019; Balogun, Jarzabkowski, & Vaara, 2011; Friesl & Silberzahn, 2017). For example, a prior study of MNE restructuring reveals the nuances of organizational negotiations to resist and legitimize a shutdown (Erkama & Vaara, 2010). But in situations of subsidiary death, negotiations to resist become redundant as the closure decision is final.

Organizational death is an unfolding process of transitioning towards closure. Prior studies highlight how this transition can be influenced by actions from leadership to restore motivation among organizational members (Crosina & Pratt, 2019; Harris & Sutton, 1986; Sutton, 1987). Next, we elaborate on both emotional experiences and leadership actions as they relate to organizational death, making conceptual links to knowledge transfer processes.

#### Emotions and subsidiary death

The literature on organizational death emphasizes that it is associated with loss. For an employee, it has parallels to the death of a loved one as it means the loss of a major social arena where people would have spent much of their time – one that defined part of their personal identity – in addition to the loss of mutual obligations (Harris & Sutton, 1986). Organizational death also means job loss in terms of an involuntary termination of employment (Crosina & Pratt, 2019). It is important to note that, while each employee has individual-level emotions (i.e., transient feeling states; Maitlis, Vogus, & Lawrence, 2013), collective-level emotions are common. This is because feeling states are shared across an organization; studies of organizational death note that the emotional response is similar to that of a process of grieving (Blau, 2006, 2008) or mourning (Crosina & Pratt, 2019) experienced en masse because a large number of employees are affected. For example, Blau (2006: 13) notes that feelings during organizational closure include an understanding that there are “no survivors” and all employees are “victims.” Especially during the initial grieving stage (Blau, 2006, 2008), feelings such as denial, anger, and depression can lead to demotivated and destructive work behavior by employees (Blau, 2007). While there is a lack of insight into subsidiary death, these prior studies on organizational death led us to expect that demotivation could occur among subsidiary employees^2^ – thereby reducing their willingness to engage in knowledge transfers.

#### Leader action and subsidiary death

Some studies of organizational death focused on the behavior of organizational members generally (Crosina & Pratt, 2019; Cunningham, 1997; Walsh & Bartunek, 2011, 2012). However, our focus is specifically on the behaviors of subsidiary leaders in response to changes instigated by headquarters (HQ) because leader actions and managerial interventions have a considerable influence on the unfolding process of organizational death. Sutton (1987), for example, finds that the decision of organizational members to stay connected or distance themselves from the organization during the closure process is influenced by how leaders orchestrate the transition towards organizational death. Surprisingly, leaders who manage the process well can ensure increased (or at least a similar amount of) effort, quality, and productivity from organizational members rather than less. This focus on leader actions also aligns with findings on how organizational context, which is shaped by management, influences knowledge transfers in MNEs (Davenport & Prusak, 1998; Gao & Riley, 2010; Minbaeva, 2007). While such a view on organizational death may be criticized for treating grief as a managerial problem to be resolved (Bell & Taylor, 2011), it enables us to understand how leaders can influence the course of subsidiary closure.

Given likely demotivation among employees, prior literature points out the need for leaders to re-instill motivation. As Harris and Sutton (1986: 24) note, “restoring goodwill is a crucial step in ensuring that employees will continue to do their jobs and will not engage in counterproductive behaviors.” However, restoring motivation among organizational members is not a trivial task because the efficacy of many common organizational levers for encouraging motivation is eroded in situations of subsidiary death. In terms of extrinsic motivation for knowledge transfer, rewards such as job security and promotion are now irrelevant (Harris & Sutton, 1986). In terms of intrinsic motivation for sharing knowledge, the efficacy of key mechanisms is also negatively impacted. For example, future reciprocity in terms of the receiver sharing their knowledge is no longer relevant to subsidiary employees as they will soon exit the MNE. During organizational death, employees may also distance themselves from the organization (Sutton, 1987). If subsidiary employees no longer identify with the MNE, the behavioral norm of cooperation during knowledge transfers (triggered by a shared corporate identity, as noted in Kogut & Zander, 1993, 1996) becomes less relevant. Given that studies of knowledge transfer in MNEs and organizational death offer little insight into how subsidiary employees react in situations where the activities of their unit are being relocated or how knowledge transfers to other units is provided for and achieved, we ask: how is knowledge transferred during a subsidiary closure and relocation?

## METHODS

### Research Design and Case Description

Given the absence of insights on knowledge transfers during subsidiary closure and relocations and how it can be managed, we chose a case study approach as it is suitable for building and extending theory (Lee, 1999). Specifically, we undertook a longitudinal case study of Gamma, the Irish subsidiary of a U.S. multinational in the information and communications technology industry. The detailed investigation of a single case offers explanatory power by providing new insights into a phenomenon that is both difficult to access and observe (Balogun et al., 2019). The longitudinal study of a single organization is particularly relevant in the MNE context to understand the complexity of an organizational process (Mees-Buss, Welch, & Westney, 2019; Stendahl, Schriber, & Tippmann, 2021), and has also been used for the study of organizational death (Crosina & Pratt, 2019).

We chose Gamma as it is a theoretically salient case (Yin, 2014) of knowledge transfer triggered by a decision to close a subsidiary and relocate its activities within the MNE. After the closure announcement, Gamma underwent a year-long process of dying that ended with its closure. Aligning with theory on organizational death (Sutton, 1987), the decision to close the subsidiary was unambiguous and final. The case is also theoretically salient because it involved the relocation, rather than termination, of all activities – including the transfer of tacit and organizationally complex knowledge. Building on relationships from prior research projects with Gamma, we were given the rare opportunity to observe the process unfolding over time (Pettigrew, 1990), despite the organizational and personal sensitivities involved.

In terms of an overview of the case, several points provide context for our findings. To start, Gamma’s activities were in a declining business of a successful, diversified corporation. While Gamma was a high-performing subsidiary with a strong track record and credibility (it had been operating for over 20 years), it was one of the first victims of an MNE-wide consolidation and cost-cutting effort to reduce the number of sites worldwide.

Next, Gamma employed more than 500 people in competence-creating and competence-exploiting mandates (Cantwell & Mudambi, 2005) at the time of the closure announcement. Some of the activities involved business-critical competences that can be classified as subsidiary-specific advantage (Rugman & Verbeke, 2001). The range of activities included new product development, engineering, manufacturing, marketing, supply chain management, and business intelligence. As most of those activities were still critical despite the overall decline in the business, they had to be relocated. All activities needed to be transferred either to other internal units in Europe, Asia, or headquarters in the U.S., or in a few instances to external outsourcing providers. Our study focuses on knowledge transfer to internal units of the MNE as this was the predominant knowledge transfer process, allowed for theorization on intra-MNE knowledge transfers, and related directly to our research question.

In terms of the workforce, it is important to note that most employees had been with the company for many years, including many who had joined Gamma when it was initially established. This meant that employee attachment and commitment to the organization tended to be high. In terms of skill profile, Gamma was led by experienced subsidiary managers who, in the past, had successfully realized many entrepreneurial initiatives; these initiatives had transformed subsidiary activities and evolved its mandate (Birkinshaw, 1996; Tippmann et al., 2018; Tippmann, Sharkey Scott, & Mangematin, 2012). Trust and confidence in the subsidiary leadership team was thus high. While many employees were experienced and well educated with third-level and even doctoral degrees, there were also some low-skilled employees working as operators in manufacturing.

Finally, the unemployment rate in the local economy was very low at the time of subsidiary closure. The existence of other job opportunities meant that there was a risk of employee loss before the completion of knowledge transfers. Moreover, all employees were entitled to a statutory redundancy payment under local employment law, and Gamma offered additional redundancy payments based on length of service. Despite job loss and an uncertain future, this redundancy payment gave every employee short-term financial security. Overall, as dictated by employment law in the European Union (E.U.), it should also be noted that redundancy payments are typically more generous in E.U. countries than in other regions.

### Data Collection

We combined real-time and retrospective data collection techniques to capture the entire closure process. As typical for case studies, we built our dataset around interview data complemented by archival data. We completed a total of 28 semi-structured interviews: eight prior to the closure announcement, eleven during the unfolding process of subsidiary closure, and nine post-death. Of these, we interviewed five people twice. All three authors engaged in the fieldwork and were therefore intimately familiar with the data.

The interviews prior to closure were part of a previous study and ensured we were intimately familiar with the preceding mandate changes of the subsidiary, its local site leadership, and the wider business context (Tippmann et al., 2018). This familiarity and prior relationship gave us the necessary credibility to gain real-time access to Gamma to investigate such a sensitive topic as it unfolded. It also promoted openness among respondents, who were asked to share subjective perceptions and feelings. Moreover, the purpose of including the prior interviews in this study was to inform the analysis of subsidiary identity before the closure announcement.

Additional waves of interviews occurred shortly after the closure decision and lasted 2 months; a further phase of data collection took place 12 months after the closure announcement (towards the end of the closure process) and lasted another 3 months; and the final phase of data collection occurred after the closure was realized and lasted a further 3 months. The time gap to the post-death interviews allowed us to probe deeper into the themes emerging from our initial analysis – a key strength of the case study approach.

We conducted interviews with managers at different levels of seniority (including the General Manager, Directors, and lower-level managers) and from different functional areas (e.g., R&D, engineering, and business intelligence). Interviews with senior managers afforded the opportunity to examine the overall strategy behind the closure, including management of the headquarters-subsidiary relationship during a time of disruption. Additionally, we included interviews with lower-level managers in our study, allowing us the opportunity to gain insights from respondents more familiar with front-line employee concerns and emotions. These insights extended into operational aspects regarding the relocation of activities and employee engagement. The inclusion of managers at different levels of seniority combined with respondents from various functional areas ensured a diversity of perspectives from across the subsidiary. While Gamma made employees redundant in phases staggered throughout the closure process, most of our informants stayed until nearly or to the very end. This provided a comprehensive picture of the entire process. Also, one of our informants opted for an internal company transfer to HQ that itself received some of Gamma’s activities, providing an opportunity to gain insights from a receiving unit on knowledge transfers.

In terms of the interviews themselves, we used a simple numbering system to preserve the anonymity of our interviewees. Interviews lasted between 30 and 100 minutes. With the exception of five interviews conducted prior to the closure announcement (where detailed notes were taken), all interviews were recorded and transcribed verbatim. Pre-closure interviews focused on the mandates of the subsidiary, its role within the corporation, and strategic activities related to role changes. These interviews attuned us to the fact that the subsidiary’s mandates, while making a substantial value-added contribution to the corporation, were in a declining business and the subsidiary had already experienced a decrease in its manufacturing activities. It further became evident that the subsidiary had a well-established identity with a strong track record and credibility within the corporation. In interviews during and after subsidiary death, we asked about knowledge transfer activities during subsidiary relocation and how any closure challenges (if apparent) were managed. Already in the initial interviews, we noticed that the feelings and sentiments of employees had played a critical role during the subsidiary death. Our approach aimed to embed emerging insights with subsequent inquiry to include prominent factors that emerged (Reuber & Fischer, 2021). We therefore sought to capture more insights on emotions and their influence over the unfolding process as data collection progressed; relevant questions from the interview guide are included in the “Appendix”.

In terms of archival materials, we collected more than 140 pages of relevant data from media coverage and materials from the national development authority, and secondary data in the form of a report prepared for a government agency on the shutdown. This data was important as it provided not only background information on Gamma and its closure, but also real-time evidence that captured the perceptions and feelings of employees between the closure announcement and the final closure a year later. The report for the government agency was based on an externally conducted, detailed interview-based data collection and therefore included in-depth insights. We used data from all the archival materials to triangulate interview data and verify the timeline of events (Cuervo-Cazurra, Andersson, Brannen, Nielsen, & Reuber, 2016; Flick, 1992).

### Data Analysis

Using interview and archival data, we compiled a case history for Gamma’s closure. In terms of our overall analysis approach, while inductive reasoning pre-dominated, we used deduction to locate our phenomenon within an existing body of literature (Mantere & Ketokivi, 2013). Based on theories of organizational death (Crosina & Pratt, 2019; Sutton, 1987) and post-death organizing (Walsh & Bartunek, 2012), we expected that the emotions of subsidiary employees and actions taken by leaders would play an important role in subsidiary death. Our initial analysis was therefore attuned to identifying issues and instances related to those two aspects. Moreover, we were attuned to previous findings that processes of organizational death involve distinct phases (e.g., Sutton, 1987). Sensitive to the possibility of temporal differences, we noticed early that the feelings, leadership activities, and identity of the subsidiary evolved over time.

Following this initial analysis, we sought to identify the specific phases of subsidiary death. Using the temporal bracketing technique (Langley, 1999), we decomposed the flow of leadership activities, employee feelings, and subsidiary identity into sequences punctuated by a change in the impact on knowledge transfer. This led us to identify two phases of subsidiary closure that impact knowledge transfer: the *break in cooperative behavior* phase and *reinstated cooperative behavior* phase. Next, we used inductive analysis techniques (Corbin & Strauss, 2008) to analyze employee feelings and leadership activities in detail. We started by creating first-order codes.

As typical for studies that seek to understand emotions in organizational processes, we analyzed the emotional experiences reported verbally by individuals in the interviews and archival data (as opposed to a direct display of employee emotions in real time). This data involved retrospective accounts in addition to accounts of emotions experienced at the point of data collection. While this data largely included the interviewee’s perceptions of the emotions of others (Kouamé & Liu, 2020); it also included emotions of the interviewee – as the managers interviewed were also subsidiary employees affected by the closure. As we sought to identify the collective character of the emotions experienced within a cohort of employees (so-called shared emotions), we triangulated what different interviewees reported as witnesses of expressed feeling states to validate the plausibility of those perceptions (see Vuori & Huy, 2016). As we had interviewed subsidiary managers of various levels of seniority, the data on perceived emotions by others captured a large base of subsidiary employees. Overall, triangulation helped minimize the risk of impression management by hiding true feelings and forgetting affective responses (Kouamé & Liu, 2020).

As organizational death is generally an emotion-laden experience, we find that descriptions of emotions and emotional reactions were frequent and explicit in our data; these tended to have a moderate-to-high level of activation (similar to prior studies; see Crosina & Pratt, 2019; Walsh & Bartunek, 2011). Attending to the valence of the emotions, we noticed that some were of a negative character while others were positive in that they either impeded or enabled action in terms of the necessary steps to transfer knowledge from Gamma to other units. The valence is captured in the aggregate dimension of *emotions*, i.e., the prevailing shared feelings of subsidiary employees, differentiating between emotional barriers (feelings that inhibit positive progress in transferring knowledge) and emotional enablers (feelings that facilitate positive progress in transferring knowledge).

Additionally, we developed the aggregate dimension for *subsidiary leadership practices* related to the activities of subsidiary managers and their efforts at enabling and executing knowledge transfer. We noticed that many of our first-order categories spoke to subsidiary leaders trying to support and influence the employees in forming an interpretation of ongoing events and the impending closure. This drew our attention to sensegiving (Gioia & Chittipeddi, 1991; Maitlis & Christianson, 2014; Maitlis & Lawrence, 2007), which captures efforts made to influence the sensemaking of others (Gioia & Chittipeddi, 1991). We also noticed that many of the subsidiary managers’ activities were aimed at influencing the emotional experience of employees (including lower-level managers) and designing different incentives to motivate knowledge transfers.

Finally, we advance the aggregate dimension of *subsidiary identity dynamics* to capture how subsidiary identity evolved during the closure process. Similarly to how organizational identity captures the essence of an organization (Albert & Whetten, 1985; Ashforth, Schinoff, & Brickson, 2020; Kreiner, Hollensbe, Sheep, Smith, & Kataria, 2015), subsidiary identity refers to a socially constructed perception of a subsidiary’s core characteristics that is broadly shared by subsidiary members: it is “their deeply rooted assumptions about who we are and can be” (Gioia, Patvardhan, Hamilton, & Corley, 2013: 127). Similarly, a MNE meta-identity refers to an understanding of the organization as an "integrative structure" that "binds together" sub-identities (Pratt & Kraatz, 2009: 387) and is “central, enduring, and distinctive to organizational members in the various units” (Fortwengel, 2021: 1070). Prior research shows that a subsidiary identity may sit outside this MNE meta-identity, for example, in the case of a recent acquisition (Clark & Geppert, 2011), while a subsidiary can be seen as “sharing” the MNE meta-identity if subsidiary employees feel that their unit’s identity is nested within the corporation’s meta-identity i.e., the MNE meta-identity stretches across the subsidiary. A subsidiary identity may sit within the MNE meta-identity (be “nested”) yet still remain distinct (Albert, Ashforth, & Dutton, 2000; Ashforth, Rogers, & Corley, 2011; Fortwengel, 2021; Kane, 2010) in how it blends aspects of the local and global in a unique way (Colman, Grøgaard, & Stensaker, 2022; Edman, 2016; Pant & Ramachandran, 2017; Voisey, 2010), or privileges either the local or global aspects of its identity (Vaara & Tienari, 2011).

Our analysis, which largely draws upon subsidiary managers’ perceptions, revealed that the subsidiary closure announcement fundamentally disrupted core elements of the established subsidiary identity. However, similar to the notion of a legacy identification in organization studies (Eury, Kreiner, Trevino, & Gioia, 2018), capturing how an identity maintains part of its past core characteristics within its current self-description, a legacy subsidiary identity emerged as the closure progressed. We analyzed how both the previously established subsidiary identity and the legacy subsidiary identity related to the MNE meta-identity to establish whether subsidiary employees shared the MNE meta-identity following the closure announcement. Table 1 below captures our final data structure.

**Table 1:** Data structure

First-order codes	Theoretical categories	Aggregate dimensions
Rejection of announcement/closure and employees disengage with MNE	Break in cooperative behavior	Impact on knowledge transfer
Statements that conveyed conviction that something needed to happen quickly to re-engage staff		
Descriptions that conveyed how staff were willing to engage with knowledge transfer activities	Reinstated cooperative behavior	
Statements that indicated objective of protecting and preserving company and staff interests		
Anger, frustration, discontent, shock	Emotional barriers	Emotions
Mistrust, betrayal		
Sad, upset		
Pride	Emotional enablers	
Trust, sense of purpose		
Counselling	Engaging with emotions	Subsidiary leadership practices
Reassuring		
Training and professional development	Reconfiguring incentives	
Additional financial incentives		
Reflection, inflection, and establishing a new narrative	Sensegiving for subsidiary identity	
Local leaders emphasizing tradition of site delivery		
Informal one-to-ones and group sessions to reinforce sense of “we’re all in it together”		
Established subsidiary identity based on historical performance	Disruption to subsidiary identity	Subsidiary identity dynamics
Discontinuity in subsidiary identity (due to closure announcement)		
Some continuity in subsidiary identity	Legacy subsidiary identity	
Task-focused, narrower subsidiary identity		

In terms of relationships between our constructs, we found that the emotional response by subsidiary employees, leadership practices, and subsidiary identity discontinuity were interlaced. Specifically, we found that the subsidiary identity dynamics acted as a key underlying causal mechanism. To analyze this in detail, we used a temporal sequencing technique that examined similarities and differences in constructs over time; by doing so, we moved towards a theoretical integration and a process model (Grodal, Anteby, & Holm, 2021). As an initial step in this analysis, we examined how the perceptions of the emotional responses of employees and leadership practices manifested across the two phases of knowledge transfer and how their relationship was described in the data*.* We noticed some important relationships between constructs (particularly within the second phase) as well as differences across the two phases.

To explain these relationships (George, Bennett, Lynn-Jones, & Miller, 2005; Thomas, Cuervo-Cazurra, & Brannen, 2011), we analyzed how our informants described connections between the perceived emotional responses of subsidiary employees, leadership practices, and the influence of those practices on the unfolding process (particularly in terms of enabling or hindering the completion of knowledge transfers). The logical starting point was the subsidiary closure announcement, as this was the trigger event for the emotional responses, subsequent leadership practices, and subsidiary identity dynamics that emerged. Our focus on analyzing how emotional responses and experience influenced actions aligns with theoretical arguments about cognition shaping behavior (Kouamé & Liu, 2020).

As our analysis progressed, we also noticed that subsidiary leadership practices had shaped the emotional responses of subsidiary employees at lower levels. This was evident in lower and mid-level managers making frequent reference to the importance of the subsidiary senior management actions in influencing subsidiary employee emotions. For example, lower-level manager noted how senior managers “looked genuine, they felt genuine and their emotional intelligence was very good” (Respondent 19). Moreover, we noticed that the death of Gamma was described as “successful for them as a whole” (Respondent 19), denoting a desired outcome for the corporation in terms of knowledge transfer. Our analysis therefore became concerned with identifying the relationships between our concepts that enabled this positive outcome. To strengthen the trustworthiness of our findings, we member-checked emerging themes and the emerging model with some interviewees (Lincoln & Guba, 1985).

The final outcome of our analysis was a process model that illustrates knowledge transfer during subsidiary death; rather than offering propositions, our model presents a set of mechanisms to explain an outcome (Cornelissen, 2017). To present the findings of our process study, we opted for a conceptualized composition where the concepts are introduced first, followed by the theoretical model (Berends & Deken, 2021).

While our theoretical model is based on the study of one organization, we had an opportunity to examine transferability by investigating the closure of another long-established, primarily manufacturing subsidiary of a European MNE. Operating in the pharmaceutical industry, this subsidiary was also based in Ireland. There, over 120 employees were made redundant, and the subsidiary’s activities were largely relocated within the MNE. After examining archival data and interviewing a subsidiary leader, we found that our model of knowledge transfer during subsidiary death applied. Regardless, we elaborate on the boundary conditions and limitations of our findings in the Discussion section.

## PHASES OF KNOWLEDGE TRANSFER FOR SUBSIDIARY RELOCATION

In Gamma, all subsidiary activities were to be relocated. This demanded the transfer of existing knowledge, including tacit and business critical knowledge, to other locations:

“We needed an effective and timely transfer of knowledge. There were millions of dollars’ worth of expertise and know-how, data analytics, customer insights and so on that were controlled by key people in the organization. And we needed to transfer this over to the overseas team” (Gamma employee as quoted in government report).

In the section that follows, we describe the two phases of knowledge transfer for subsidiary relocation: *break in cooperative behavior* and *reinstated cooperative behavior* for knowledge transfer. For each phase, we illustrate the interplay of emotional responses by subsidiary employees and shifting subsidiary identity dynamics. For the second phase, we also present subsidiary leadership practices that allowed for progression towards a successful transfer of knowledge from the Gamma subsidiary to other sites within the MNE.

### Break in Cooperative Behavior: Discontinuity in Subsidiary Identity after Closure Announcement Evokes Emotional Barriers Inhibiting Knowledge Transfer

As the first phase, *the break in cooperative behavior*, captures the response to the announcement of subsidiary closure (that is, the point at which employees were informed that the subsidiary would be ceasing operations) and the subsequent unwillingness among employees to accept this decision and engage in knowledge transfer activities. While prior studies have established that organizational members may be demotivated and disengage during organizational death (Blau, 2007; Harris & Sutton, 1986; Sutton, 1987), our results show the extent of this issue during subsidiary death. In particular, our findings highlight the severity and immediate implications for the MNE as knowledge transfer was inhibited. Respondents noted how the subsidiary closure announcement compromised their willingness to cooperate:

“Here’s a site that has just been told that look, they’re going to be closing down in a year. And oh, by the way, we want you to transfer your knowledge to these people over here, over there. What’s the motivation, the impetus to be able to make sure that that is going to happen?” (Respondent 10).

To illustrate further, we elaborate on the intricacies of this initial phase and provide additional empirical evidence in Table 2.

**Table 2:** Break in cooperative behavior

Category and first-order code	Illustrative data
*Break in cooperative behavior*	
Rejection of announcement/closure and employees disengage with MNE	“It was laid out to the business very, very clearly. People were quite vocal at the announcement that, if you want this transfer to happen, and you mess us over, it will not happen.” (Respondent 16)
	“After the dissent in the first week or two, there was a lot of tension. Fighting over the terms, an employee counsel was set up.” (Respondent 15)
Statements that conveyed belief that something needed to happen quickly to re-engage staff	“One of the smartest things the Ireland management team did locally, and it is now seen as a best practice in the company, was to say, ‘Okay, over the next year, we know that you folks are being made redundant, we’re going to do everything in our power to put you in the best position possible to find employment once you leave [Gamma]’. … And the people in Ireland saw that significant investment as more goodwill if you will in making this whole transition happen as smooth as possible.” (Respondent 10)
	“The additional follow up mechanisms happened very, very quickly in that a consultation group was formed – very, very quickly formed. How do we support our staff in terms of venturing out to this new world, and what are the things that they need to be aware of? So, for example, what support mechanisms do they need for going and looking for jobs?” (Respondent 18)
*Emotional barriers*	
Anger, frustration, discontent, shock	“There was a lot of anger in the room. I mean real anger... people were just angry at being let down... I think they were just really angry at the suddenness after all they'd done and after all the programs for employee engagement. This is hypocrisy.” (Respondent 15)
	“Some departments were shell shocked, because they thought they were completely protected... they were just caught off guard, because they thought they were in a bubble... a number of departments were caught off guard.” (Respondent 14)
Mistrust, betrayal	“It had been certainly concealed from people that this [closure announcement] had obviously been in planning for quite some time, and there was a level of orchestration and polish around the delivery of the message that also didn’t appeal to people. There was a level of deceit associated with it.” (Respondent 17)
	“For the last 20 years you have trusted them, and they have trusted you. And then you walk in one day, and your trust is broken.” (Respondent 14)
Sad, upset	“I think in the [Gamma] Ireland site, most people have been there for more than 10 years. And then we probably have 20/30% of people that have been there 20 years. So, it’s been quite a traumatic thing to happen.” (Respondent 11)
	“People were in shock. People were very upset.” (Respondent 17)
*Disruption to subsidiary identity*	
Established subsidiary identity based on historical performance	“There was a sense that we've had ups and downs, we've had successes and failures where we were making pitches for projects or programs and people knew, when something was successful, they knew the effort that went into it. It wasn’t like we only knew each other for a while. We'd shared experiences of success for many, many years and some failures along the way as well. They were all shared experiences.” (Respondent 17)
	“We took on more difficult projects, we took on projects with more dependency, we took on projects which required more finesse. And those projects came to Ireland, not because we were the cheapest, not because we were necessarily the best coders, but because we had a development system that enabled things to be done quickly, at high quality. And we were good at managing software projects and delivering software projects … we had a great software program that was built from Ireland.” (Respondent 1)
Discontinuity in subsidiary identity (due to closure announcement)	“Right after that announcement, and if that’s done wrong, which it was in [Gamma], you’ve basically multiplied the difficulty [of knowledge transfer] by 10 because now you’ve lost the loyalty of the employees, you’ve lost the goodwill, it's been squandered. But local management are going to be held accountable nevertheless for delivering on that [knowledge transfer]. So they have their work, they’ve got hundreds of disaffected employees, and they have to turn around now and ask them to work professionally when they really don’t want to… There was a sense of betrayal because we made billions for this company, literally billions in margin over the 20 years. It was enormous, it was measured in the billions. They [employees] couldn’t understand why, having done everything that was asked of us, that the company was no longer fulfilling its side of the social contract.” (Respondent 16)
	“Some people would be psychologically affected by it, that they’ve lost their workmates, their teams, their people.” (Respondent 15)

#### Emotional barriers

The closure announcement signaled inevitable job losses. Emotional barriers, which are defined as strong negative emotional reactions among subsidiary employees that inhibit knowledge transfers, then manifested. These barriers included feelings of (1) anger, frustration, discontent, and shock; (2) mistrust and betrayal; and (3) sadness and upset.

Only very few senior managers of the subsidiary were aware of the impending closure prior to its announcement. Most employees, including many in subsidiary leadership roles, were unaware and unprepared. This caused anger, discontent, and shock. One respondent noted that “some departments were shell-shocked, because they thought they were completely protected…they were just caught off guard” (Respondent 14). Employees were shocked at the scale and magnitude of the announcement, which effectively meant that all operations (not just some activities) at the subsidiary would cease: “it was not a surprise that they were downsizing. It was a surprise for a lot of us that they took the nuclear option, which is you pull the plug” (Respondent 18). Media reports focused on the magnitude of the closure, where over 500 people would lose their job (National newspaper), and how “some families now are facing the loss of two salaries. They are not ‘impacted’ by this closure; they will be crucified by it” (National newspaper). In addition to a feeling of shock, employees were also upset, angry, and felt betrayed: “people were in shock, people were very upset…There was a level of deceit associated with it” (Respondent 17).

These emotions represented barriers to knowledge transfer and the relocation of subsidiary activities because they resulted in an unwillingness among employees to accept the subsidiary closure decision, including a denial that the decision would be enacted. One respondent reflected:

“If I look at the first few weeks, it was anger. How could they do this to us? We’d been such a high performing business as a site for so long; they'd made a wrong decision. At the start, people were probably thinking they’ll reverse this decision; they know they’ve made a mistake; or this is wrong, and nobody could understand it. You could even see senior [subsidiary] leaders questioning: Why are they doing this? This isn’t the right decision” (Respondent 19).

Given strong emotional barriers, subsidiary leaders realized that it would not be possible to attempt knowledge transfer and a relocation of subsidiary activities at that time. Indeed, there were tremendous levels of employee disengagement and loss of commitment:

“They [corporate leaders] made a decision to close down and that changed everything. That meant all of that effort of fighting your battle, if you like, ended…they [subsidiary employees] just weren’t motivated positively to do it [transfer knowledge]” (Respondent 16).

Another respondent similarly described this lack of motivation to engage in knowledge transfer: “some people have the attitude of ‘let them fend for themselves. I’m not going to help them; they’re taking my job’” (Respondent 19). One manager further highlighted the extent of damage that the announcement had caused:

“The announcement itself is a pivot point that sets the tone, and after that, we were in a very unfortunate situation where it was damage control… some of them [corporate leaders who made the announcement] just wanted to literally run to the airport, because they knew they had a disaster on their hands” (Respondent 16).

With worrying levels of disengagement and a loss of commitment among subsidiary employees, emotional barriers made it impossible for the subsidiary to successfully transfer knowledge and relocate activities. Further, for many employees, the closure announcement prompted disruption to an existing and well-established subsidiary identity and raised many questions as to why the subsidiary (and the roles within it) were now rendered obsolete (Bell, 2012; Harris & Sutton, 1986; Sutton, 1987). We detail these dynamics below.

#### Disruption to subsidiary identity

Shortly after the subsidiary closure announcement and the erection of immediate emotional barriers, we observed a discontinuity in subsidiary identity. Discontinuity is defined as a disruption to the existing subsidiary identity experienced by employees, wherein the “character of one’s identity” (Eury et al., 2018: 836) was misaligned with prior understandings. Prior to the announcement, the subsidiary identity of Gamma was one of a distinct, high-impact site with a long history of performance. This identity had formed based on a history of publicized, well-known achievements of the subsidiary: “we always had products on the go, always had new ideas, and we had good working relationships with the U.S. because of our business contributions. It was well earned because we seemed to be a high-impact site” (Respondent 17). Managers highlighted how the subsidiary had “found our niche for the last 10 years…we were doing a great job and getting great recognition because of the revenue it was generating” (Respondent 11). This was coupled with an expectation of sustaining the subsidiary in the future by “developing the infrastructure that made us valuable, that made us less expendable” (Respondent 15). It is important to note that such descriptions of subsidiary identity as “a high-impact site,” “business contribution,” and “finding a niche” all refer to the subsidiary as part of a global business, thereby positioning the Gamma subsidiary within that corporate network. This also means that the subsidiary identity was seen as nested within the MNE’s meta-identity as a global organization (Fortwengel, 2021), and that subsidiary employees of Gamma shared the MNE meta-identity (Kogut & Zander, 1993; Pant & Ramachandran, 2017).

The closure announcement was delivered by corporate staff external to the subsidiary. It involved a statement that the site would be closed as a global cost-saving measure and that employees would be made redundant over a 12-month period with an initial cohort leaving within 5 months. Closure and relocation of subsidiary activities to other sites was inconsistent with the established subsidiary identity and threatened the perception ingrained among Gamma’s employees of the site as a high-impact and high-performing subsidiary of the MNE. It contradicted a strong subsidiary identity built upon previous achievements and historical performance:

“Why is the site shutting down? Why am I losing my job? Why can’t I come to work anymore? So, you’re being very close to an individual’s personal life in a job they could have been in for twenty plus years. And they gave so much to the company. So, there’s a huge emotional connection with that community, that organization. You are part of a community, and they have just broken your trust” (Respondent 14).

This interplay between strong emotional responses and the perceived inconsistency with an established subsidiary identity aligns with previous studies of organizational death (Harris & Sutton, 1986). Additionally, we observed how disengagement with the MNE meta-identity directly impacted on the willingness of employees to engage with knowledge transfers. Respondents described how “it became like a siege mentality… you stopped caring about corporate at that point, because all you trusted were the people you were able to talk to face-to-face, and who were genuine” (Respondent 19). The challenges for knowledge transfer, in particular for the transfer of tacit knowledge that requires extensive communications, were well documented: “it was pretty hard to get people that would give that kind of commitment in a shutdown situation” (Respondent 13). Next, we describe the second phase whereby the interplay of subsidiary leadership practices, emotional responses, and subsidiary identity dynamics become apparent.

### Reinstated Cooperative Behavior: Regaining Commitment of Subsidiary Employees to Enable Knowledge Transfer

The second phase, *reinstated cooperative behavior*, captures efforts by management to engage with subsidiary employees and regain their commitment in order to realize knowledge transfers. Local managers recognized the immediate need for greater efforts to re-engage staff to enlist their support in the closing of the site:

“You need to develop an anchor or a fishhook to bring them [the employees] back – throw them a life buoy or a life vest – because they can completely emotionally switch off and still end up walking out the door legally entitled to a [redundancy] package. But they don’t have to do anything. So, you need to somehow get them back” (Respondent 14).

To reinstate cooperative behavior, subsidiary managers adopted three local leadership practices: (1) *engaging with emotions*; (2) *reconfiguring incentives*; and (3) *sensegiving for subsidiary identity*. These subsidiary leadership practices helped to reinvigorate a willingness to cooperate in knowledge transfer activities. We elaborate on these mechanisms next and provide additional data extracts in Table 3.

**Table 3:** Reinstated cooperative behavior

Category and first-order code	Illustrative data
*Reinstated cooperative behavior*	
Descriptions that conveyed how staff were willing to engage with knowledge transfer activities	“There was the exceptionally high level of professionalism from the Ireland site when it came right down to it. People wanted their knowledge to live on, they wanted their partners [receiving sites] to be successful, and it was important to them to do a good job. And that drove a lot of the motivation.” (Respondent 10)
	“Of the 450 odd employees impacted by the Ireland exit, I would say easily 250, 300 were directly involved in transferring knowledge. And that may be as simple as phone conversations with the recipient, or it could be manufacturing guys on the line bringing teams around, showing them, training them. So, it was very much an employee led activity; employees were very much involved all the way... that’s the only way it could have been successful because the knowledge is certainly not centralized, it’s very broad.” (Respondent 3)
Statements that indicated objective of protecting and preserving company and staff interest	“We were under obligation to protect the company, its brand, its revenue, its profitability, and its assets. So, wearing the company hat on one side – and there’s a whole load of work that was happening to preserve that, and the second was about preserving and protecting the interests of the employees.” (Respondent 16)
	“People overall were very mature, very proactive, wanting to make sure that they did a good job of handing over the remaining activity. There were very few people that were disengaged. I could probably count them on one hand. To be honest, I think part of that is due to the way in which [Gamma] treated people. We went through extensive effort around giving people the opportunity to upskill, we developed a lot of training opportunities for people, we had subject experts coming in to provide training in lots of different areas.” (Respondent 12)
*Reconfiguring incentives*	
Training and professional development	“We agreed at the very start of the consolidation program that we'd have a form of a social contract where we would focus on people’s wellbeing, education, training, financial advice, and put a very, very good suite of support programs in place. In turn, they [subsidiary employees] would support the business in terms of transferring programs.” (Respondent 5)
	“We supported everyone with training programs, education programs. We had a wellness program. We had parties, we had farewells. We had a very comprehensive program to make it a viable experience for people over a year and set them up for success.” (Respondent 17)
Additional financial incentives	“This is where we are. We still have 9 months of work that we need to do. A chunk will involve things that you might not like seeing, which is tearing down the machines and shipping them over to the other site. That’s the nature of the beast. The company is going to support you between now and then in certain ways. In return, what the company’s looking for is that you will also support them in terms of helping with knowledge transfer or doing transfers over a period of time… there were incentives included in that to make sure that people were motivated and to make sure this worked.” (Respondent 6)
	“There were incentives put in place for our site for people, so that people would remain engaged and do a good job in transferring that knowledge.” (Respondent 12)
*Engage with emotions*	
Counselling	“Managers got plenty of support from an emotional point of view… Not everybody would openly talk about it [their feelings], but plenty did… That’s part of any therapy, that people see other people break down with that. Maybe they think they’re sad, then they see somebody stronger. Or they thought they were stronger, [but] broke down in a room full of people.” (Respondent 14)
	“Everybody could get one on one counselling sessions… we didn’t actually advertise the extent to which we were doing that upward [to headquarters management], because you don’t invite trouble.” (Respondent 16)
Reassuring	“The important thing I think in all of that was that you separate the strands of emotion and you address them all separately, right. So, the fear and anxiety about the future and their career, you get financial advisors in, which we did to help everybody on a one-to-one basis, figure out their money, how are they going to make ends meet. Anxiety around careers: a big drive on that, to say, hey, listen: you think you’ve nothing, no qualification? Of course you have. And then, because people totally underestimate their own worth and what they can bring to a company. So, you bring employers in, and you bring recruiters in, and you do all sorts of stuff that helps people believe that they do have value, that they do have something they can bring to the market. And so, we did a lot of that.” (Respondent 16)
	“I suppose there’s a curve of emotions that people go through… you’re still trying to do the right thing for them, be as flexible, ask them how they’re getting on, mental health, all that good stuff, it's important. I think people go through cycles like grief, people deal with it differently.” (Respondent 4)
*Sensegiving for subsidiary identity*	
Reflection, inflection, and establishing a new narrative	“People internalized that this business is not doing well, and, to an extent, it was also reinforced by the local management. Explaining why a company is pulling out of a country can be best understood by looking at the macro financial picture and the macro business picture. So, we did spend a lot of time on that. We explained to people what was going on and why things were happening… it’s really a situation where authentic leadership is absolutely crucial and credibility is crucial.” (Respondent 17)
	“People could see that while the corporation had made a decision to cancel or close the site, the Ireland site and the leadership team had done a good job of securing, I suppose, extra recognition for the fact that people would have to do this in a context where they are knowingly losing their job and effectively training other people to do their job.” (Respondent 12)
Local leaders emphasizing tradition of site delivery	“We had spent years developing the infrastructure that made us valuable, that made us less expendable... We've done a great job, we've always done higher value add, higher development activity, we got out of manufacturing. [Plant Manager] actually pulled us out of manufacturing ahead of the curve. So, we've done an amazing job to still be here. But now, here we are, so let’s continue and let’s do it [subsidiary closure].” (Respondent 15)
	“The Ireland team is known for being quite aggressive. They’re known for delivering on commitments... [we] were able to get ownership for things like business insights and forecasting, we had a marketing team, we had a competitive intelligence team that we developed. So, all of those were started with seedlings of expertise and then they gradually grew.” (Respondent 7)
Informal one-to-ones and group sessions to reinforce sense of “we’re all in this together”	“We agreed to give people time. Communicate and have sessions openly talking about what has happened. If anybody needs to come to talk, then we’re here. Any questions at all, come back. Even if it was silence in the room, let people vent their frustration and what they’re frustrated at because that’s half the battle.” (Respondent 14)
	“Everyone rallied together and everyone helped each other out at that initial time anyway because we knew we were all in the same boat.” (Respondent 19)
*Emotional enablers*	
Trust (in local leadership)	“Because everyone was being let go you felt okay, what you're saying [local management] is genuine and I trust you. You also knew that they [local management] were putting your interests ahead of their own a lot of the time, or that’s what it felt like because a lot of what they were doing was trying to help you. You knew they needed to help themselves as well, but it felt like they were putting the employees ahead of themselves.” (Respondent 19)
	“It was the local management that stepped up, and the corporate [managers] either did not have visibility or interest in driving all the touchy-feely stuff. They didn’t really care whether or not the local company arranged training in new careers, exit interviews, training. We did a big career fair. If it was down to the company, I'd say they wouldn’t have allowed that because we spent an arm and a leg on it.” (Respondent 16)
Sense of purpose	“There was an enticement, for the want of a better word, as many people as possible wanted to stay until the end purely because there was a body of work that we needed to complete out, and there was a project handover that we needed to complete with some of our west coast folks [receiving sites in U.S.]. And that was inclusive of tacit knowledge and also transfer of assets.” (Respondent 18)
	“There’s ways to do a shutdown, [but] when you’re part of it, it's quite an emotive topic, yes. I suppose the incentives were one piece, the financial incentives do give a bit of a blanket to people and give them a little bit of a justification in their own minds why they’re doing it. But they’re not the only thing...we had an amazing engagement model here where our engagement levels for the employees were quite high… we leveraged that engagement model and used that for the knowledge transfer and the site shutdown.” (Respondent 8)
Pride	“People took pride in what they did, and they were very proud of their work. So, when they were asked to transfer the knowledge, they felt it was a good opportunity... this is a way for me to wrap up everything that I've done, be proud of it, present it, and show the people that I'm a strong person, a strong employee, and I've done good work… It was like their baby. They'd built it from the ground up, so they wanted it to be successful.” (Respondent 19)
	“There was that real sense of wanting to do the best that we could from the engineers up. It’s hard to explain, and I don’t know if any other people have referred to that as well. But I would say 95% of people were fully engaged in the process after they had picked themselves up.” (Respondent 11)
*Emotional barriers*	
Sad, upset	“People were very upset that they were going. The sadness thing is something that kind of came again and again because they [employees] had different dates for when people were leaving after they finished training somebody else in, or they finished up a line they'd leave. So every month or so a whole bunch of people would leave, and they'd be saying goodbye in some cases to people they had worked with for 20 years.” (Respondent 16)
	“There's that sense of dealing with those emotions of letting people down. Personal failure that this is happening on your watch and then just dealing with the uncertainty of where am I going to get a job? Have I got the skills? Each and every one of us has to go through that cycle ourselves.” (Respondent 17)
*Legacy subsidiary identity*	
Some continuity in subsidiary identity	“One of the things that we [Ireland R&D team] had done really, really well for 10 or 15 years: we had a R&D charter, we were able to push programs very aggressively. We were good at measuring the risk and keeping things moving in an aggressive nature. We were handing over to the R&D site in [other subsidiary site]; they didn’t have that… it was a strange dynamic because the team, even though they were losing their jobs and getting very close to an end date, there was still an element of we can do this better than the receiving site… there was nearly an uplift in spirits to a certain degree, because there was a feeling of unity in that everybody was in it together. There was also a feeling of, we've pulled this off. We've done it, we stand proud that, as a site, we've done this handover very, very successfully, and we've done a really good job, and we've done ourselves proud.” (Respondent 13)
	“The biggest thing I suppose was the leadership team on site probably signing up to it: we’re going to do this, and the employees just followed through on it.” (Respondent 8)
Task-focused, narrower subsidiary identity	“I still have a duty to provide a service… what made the knowledge transfer successful was people just put so much work into it. They really did, and I think people knew that was the finish line. It was like, once I do this knowledge transfer that’s more or less my end, what's expected of me… it actually works out better because you get that closure and you feel like you can let it go then and you can dust yourself off. Okay, it’s done and you get that piece of closure.” (Respondent 19)
	“We’re not going to drop the ball on a major product that’s in the development cycle. We're going to support the knowledge transfer, and we’re going to go out with a bang.” (Respondent 17)

#### Engaging with emotions

The first subsidiary leadership practice, engaging with emotions, captures efforts by subsidiary managers to respond to and satisfy the emotional needs experienced by employees via counselling and offering reassurances. Subsidiary managers highlighted how managing the emotions of employees was critical to building the motivation needed to complete the knowledge transfer required for relocating activities: “The hard, unfathomable piece that can potentially just run riot is the emotional responses of people…and if you don’t get that piece right, you're not going to get the other piece right [the transfer of activities]” (Respondent 16).

To engage with emotions, subsidiary managers became highly cognizant of the feelings of individuals and the ways those feelings changed over time: “Managers are dealing with individuals. They’ve got an amount of people in their team, and now they’re dealing with the emotive aspects of people being laid off...you go through that standard thing of denial – acceptance” (Respondent 6). However, even as acceptance of the subsidiary closure decision set in among subsidiary employees, managers were still faced with the challenge of re-motivating them to ensure business continuity and engagement with the knowledge transfer needed to relocate activities. One respondent expressed this challenge as:

“There’s a period of time where there’s acceptance that creeps in; ‘okay, I am now losing my job.’ The challenge of management is...there’s a body of work that we need to do to keep the business going. How do we motivate and incentivize people to be sure that they keep doing that? Because, effectively, we’re still professionals ourselves. And how do we generate that feeling amongst people to make sure that they’re still motivated and still incentivized? And that presented a challenge” (Respondent 6).

We find that subsidiary management took on a greater leadership role in engaging with the emotional needs of subsidiary employees as they were closer to the affective responses from employees compared to HQ managers. Many subsidiary managers also shared the emotional responses of their employees, which included shock, anger, denial, and upset. This gave them a better sense of what actions may be most suitable for engaging with emotions. Subsidiary managers recognized the importance of engaging with emotions by offering reassurance and emotional support:

“You’re dealing with people’s level of motivation. You’re dealing with people’s future as well, and they’re coming back to you, and they’re scratching their heads, and they’re trying to figure out their next step. And they’re looking for guidance, they’re looking for some mentorship. They’re looking for people to put an arm around their shoulder and say where’s my value? Where’s my net worth?” (Respondent 6).

To provide emotional support, subsidiary managers participated in training that better equipped them for dealing with employee anxiety. They also brought in external support, as highlighted by one respondent who noted that “every single person had a career counselling session with professionals brought in.” The same respondent also stressed a need to:

“Appreciate the anxieties [by] anticipating those anxieties and making sure you’ve taken provision. We did put extensive counselling initiatives in place. That’s very key. If people can’t function and they feel too emotionally drained to function, you’re not getting any objectives met” (Respondent 16).

Counselling sessions and engagement with the feeling states of employees helped to influence subsidiary employees' emotions. While the passage of time helped settle the emotions that presented barriers to knowledge transfer, we also observed how this practice was instrumental in reducing emotional barriers:

“It amazes me that people still come in [to work]. Some of it is that they have an element of, by coming in, you have the support network and everybody’s in the same boat – and there’s an element of support there” (Respondent 11).

Engaging with employee emotions thus helped to reassure and provide continuity – an outcome that was also observed in a prior study of organizational death (Crosina & Pratt, 2019). It further allowed individuals to repair pre-existing internalizations (Gaines, 1997: 550), or trust in local management in the case of Gamma. However, trust in corporate and headquarters management remained broken:

“I trust the Irish manager now; I no longer trust corporate, because corporate have made the decision to close the site. I think a lot of people, even people who are realists, would say they didn’t think it felt like the right thing to do... So, straight away, people said okay, we don’t care what corporate do anymore; we just care about our internal side, from our Site Manager, who was the main leader, down. You trusted them but anything above him, I don’t think anyone cared if they were involved or not” (Respondent 19).

Engagement with emotions was an activity that facilitated the reduction of emotional barriers and required continuous engagement with employees across this phase. To illustrate, as employees were being let go in batches over an extended time period, each batch of departures ran the risk of becoming a catalyst for further emotional barriers and prompted subsidiary managers to put in place ongoing emotional support. One respondent captured the emotional challenges associated with staff leaving in different phases: “the sadness thing is something that came and came again, because they had different dates for when people were leaving” (Respondent 16). Critically, lower levels of management commented on how they “have nothing but praise for the senior management. They put in a very good support structure” (Respondent 18). Similarly, the senior leaders in Gamma were cognizant of their own roles, as “so much depends on the management team” (Respondent 15), and “if Gamma had done nothing to support people, I’m sure we would have seen a very different response from individuals” (Respondent 7).

#### Reconfigure incentives to reinstate cooperative behavior

The closure announcement heightened employee self-interest, or their need to protect their individual financial well-being and to seek future employment. This shattered their motivation to cooperate in knowledge transfer on behalf of the corporation. Traditional mediums- or long-term incentives, such as promotion or job security, were no longer viable options. Subsidiary leadership therefore sought to reconfigure incentives by shifting towards progressive incentives for the immediate personal benefit of subsidiary employees in order to reinstate cooperative behavior. Specifically, these incentives included training and professional development as well as additional financial rewards to support extrinsic motivation.

In terms of training and development, this served employee interests by boosting their job market readiness. It was an incentive that employees valued, as evidenced by the level of uptake for training courses and development efforts. Out “of the 500 employees affected, 322 participated: that’s 64%” (Gamma employee as quoted in government report). The provision of additional incentives focused on skills development was configured swiftly by leadership:

“Very quickly it got into, okay this [subsidiary closure] is happening. There’s nothing we can do to change it. Now, what can we do to give people the best opportunity to maximize – whether it be training or skills – upskilling? Let’s get people on the track to get into a better position for when it does happen” (Respondent 19).

In terms of financial incentives, it was deemed necessary to explicitly offer knowledge transfer rewards to employees beyond the agreed redundancy package: “They [management] actually came out and said, ‘Look, what we’ll offer is a bonus for people who stay to transfer knowledge’” (Respondent 15). Managers highlighted how these financial incentives offered a pathway towards the transfer of knowledge. One respondent noted how “the ulterior nature of those types of things [employee supports and bonuses] would be that I’ll get buy-in from staff: they’ll stay here, and they’ll do what I’m asking them to do as part of the knowledge transfer” (Respondent 6).

This mutual arrangement acted as a mechanism for regaining employee motivation in return for the additional supports and benefits on offer: “It was reciprocated. There was a sense that you [subsidiary senior management] have stood up for us [employees], you’ve protected us, you’ve supported us” (Respondent 17). While distrust was directed at corporate managers, trust in local leaders, as a key emotional enabler was evidenced in “a really big trust circle; everyone really trusted the local team…They were able to sense what the mood was, when to discuss future training, when to discuss current situations” (Respondent 19). This trust was firmly based on shared experiences and the credibility of local senior management due to the significant efforts they took to protect employee interests. Subsidiary senior leadership, as described by those in lower and middle management roles, was highly authentic.

Leadership practices to protect employee interests played a key role as a precursor to any other efforts related to ensuring business continuity and knowledge transfer for the relocation of activities as required by the MNE. A respondent recounted this critical role as:

“We emptied every drawer that had any money in it, and it went into that [training and personal development programs] and other things … the local management understood [that] if you didn’t do these things, the company wouldn’t get its objectives met” (Respondent 16).

Collectively, the reconfiguration of incentives prompted greater employee engagement by reducing emotional barriers and adhering to both the financial and emotional needs of employees. Next, we describe how sensegiving practices facilitated a shift in the subsidiary identity dynamics.

#### Sensegiving for subsidiary identity

Sensegiving can be defined as efforts made to influence the sensemaking of others, for example by providing “a viable interpretation of a new reality” (Gioia & Chittipeddi, 1991: 443). For Gamma employees the new reality referred to the status change of an organization destined for closure, but still tasked with maintaining business continuity and associated knowledge transfer activities. We found that sensegiving practices adopted by local management supported the emergence of a legacy subsidiary identity by reinforcing the experience and reality that “everybody was in it together” (Respondent 13). Centered on communication, these sensegiving practices manifested in, for example, group and one-on-one sessions between subsidiary leaders and employees:

“A part of what my job over the last nine months was to manage people very directly through that process. It is very difficult to say to somebody in the cold light of day, ‘Yes, you are losing your job, but you have an awful lot of knowledge now, and we will go and give it to somebody else who is taking your job in a higher cost location.’ So, they were very sensitive conversations to manage” (Respondent 9).

Resulting from the significant inconsistency between the subsidiary closure decision and the established subsidiary identity of a high-performing, high-impact subsidiary with a strong track record and credibility, employees lost their sense of “who we are” as a subsidiary. Given that the subsidiary identity of Gamma prior to the closure announcement was firmly nested within the meta-MNE identity, employees experienced a noticeable disruption to their established identity. As organizational identities are socially constructed (Edman, 2016; Voisey, 2010), a new shared perception by subsidiary members had to be created to fulfil their need for a sense of purpose or legitimacy to their work (e.g., Pant & Ramachandran, 2017). This was evident in the emergence of a legacy subsidiary identity. Building on prior work (Eury et al., 2018; Kreiner & Ashforth, 2004), we define legacy subsidiary identity as the subsidiary drawing on its familiar, established identity, while reflecting the new reality of an impending site closure.

Referring to the “legacy” of Gamma provided for some continuity in that it emphasized the core high-performing attributes of the subsidiary, especially its characteristic of always delivering on its objectives. Subsidiary leaders extrapolated this core characteristic of Gamma’s identity to the closure process and the need to transfer knowledge so that a legacy subsidiary identity could manifest:

“Even since the shut-down [announcement], we have delivered over $50 million of incremental revenue ideas this year to the business. We have also primed the pump for next year with the receiving team. We have generated a list of ideas...We have gone through initial scoping and initial sizing of those opportunities with a view to trying to prime the pump for them and set them up for success for next year” (Respondent 9).

Important in the MNE context, the legacy subsidiary identity acknowledged that, while the subsidiary would die, the wider MNE would survive: “the corporation is trying to be infinite, and we are very finite within that position. They’re just trying to sustain on an ongoing basis. So, they [the corporation] will morph and twist and turn” (Respondent 9). Moreover, a clear demarcation in the legacy subsidiary identity and MNE meta-identity was evident in statements such as “They [corporate] had cut their ties … we no longer have that loyalty to the company from a corporate side” (Respondent 19). We interpreted such data as evidence of the legacy subsidiary identity no longer being nested with the MNE meta-identity and subsidiary employees no longer sharing the MNE identity.

Despite subsidiary employees no longer sharing the MNE meta-identity, the legacy subsidiary identity enabled knowledge transfers because it provided a residual connection with the traditional behavior and history of the subsidiary. In their communications, subsidiary managers reinforced this connection by stressing a return to Gamma’s history of high-performing practices and established tradition of delivering on MNE objectives. Such efforts helped achieve the objective of knowledge transfer within a specified period: “it [knowledge transfer] just becomes normalized as a new set of projects. Okay, they're terminal ones, but you can’t run away from reality” (Respondent 15). Through the emergence of a legacy subsidiary identity, sensegiving practices by subsidiary leaders re-ignited intrinsic motivation; employees now wanted to act in accord with their subsidiary’s legacy identity. This helped to reinstate cooperative behavior for knowledge transfers.

Additionally, while emotional barriers, such as sadness, were a constant across both phases, we observed how the emerging legacy subsidiary identity and associated efforts to act consistently with this identity reinforced the emotional enablers. Specifically, the legacy subsidiary identity evoked a positive feeling of pride among subsidiary employees. Evidence of this was found in subsidiary employees (including managers) reflecting on previous achievements and celebrating their collective accomplishments. Eventually, they moved towards a feeling state of closure associated with the completion of knowledge transfers as evidenced in the reflections of one subsidiary manager:

“People took pride in what they did, and they were very proud of their work. So, when they were asked to transfer the knowledge, they felt it was a good opportunity. I suppose it’s a good opportunity to get some closure on everything that you’ve done. And I think closure is a really good way to describe it, because it’s a way for me to wrap up everything that I’ve done, be proud of it, present it, and show the people that I’m a strong person, a strong employee, and I’ve done good work” (Respondent 19).

The execution of knowledge transfer activities afforded employees an avenue to demonstrate their worth, ensuring that their past and current efforts would be maintained and nurtured in the future:

“I needed to feel that the company will look after itself…it was like their baby. They’d built it from the ground up, so they wanted it to be successful. So, it was in our best interest to do the best knowledge transfer we could do. So, when the future team took it, they could keep the success going…they had such pride in what they had built” (Respondent 16).

While it may seem counterintuitive, the emotional experience of subsidiary employees towards the end of this phase was more positive than negative:

“People wanted to do their best. They wanted to say, “We’ve had a great career in [Gamma], and we’ve done some fantastic things over the last ten to fifteen years as an R&D team. We’re going to leave on a high” (Respondent 10).

Employees took this more positive emotional feeling state into the final stage of achieving the outcome of knowledge transfer during subsidiary relocation. In the section that follows, we elaborate further on this outcome.

#### Realizing knowledge transfer

As described above, subsidiary leadership practices enabled knowledge transfer both directly (via the reconfiguration of incentives) and indirectly (through their impact on the emotional experience of subsidiary employees, as well as the emergence and subsequent leveraging of a legacy subsidiary identity). At the end of the subsidiary death process, knowledge transfer objectives were realized. This was evident in statements such as “there were hundreds and hundreds of knowledge transfer trips completed across a spectrum of competencies that were needed to fulfil that overall mission, whether it be R&D technical staff, manufacturing, [or] supply chain” (Respondent 12). Another respondent observed:

“Everything that we have on active projects, everything that we have from a roadmap point of view in terms of future products, they are all still live and real and have been shared and ownership of those have been transferred” (Respondent 7).

A particular focus fell on the transfer of specialist knowledge areas, core competencies onsite, and ongoing research and development programs. Managers implemented roadmaps to ensure that milestones were set up and accountability was established at both the sender and recipient levels:

“We treated knowledge transfer as we would any project: we had a set of key deliverables, we had our milestones, we had our sign-offs. So, products that were mid-cycle, we worked within that structure…we picked a stage-gate point that would have made sense to the target point; okay, at this transition point, we will transfer accountability across for the program” (Respondent 8).

To corroborate these findings, we also gained insights from one respondent who opted to relocate from the subsidiary to HQ. He attributed the success of knowledge transfer process at Gamma as largely being dependent on how: “the company put the ownership and responsibility completely within the Ireland management chain” and explained that the organization has subsequently “used that [process] as a template for how successive knowledge transfer activities would go” (Respondent 10). At the end, the subsidiary was closed, all activities were successfully transferred, and business continuity for the corporation was maintained.

## MODEL OF KNOWLEDGE TRANSFER DURING SUBSIDIARY DEATH

**Figure 1:**
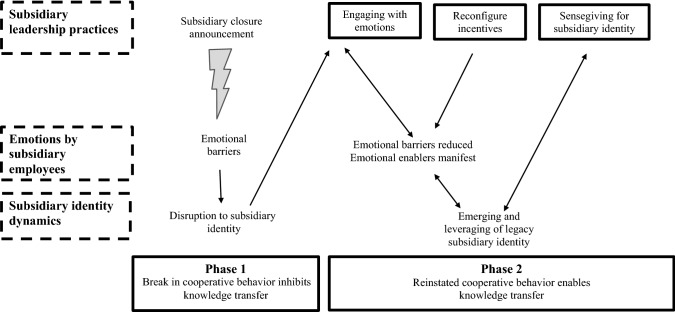
Model of knowledge transfer during subsidiary death.

Linking our empirical findings with relevant literature, we advance a process model of knowledge transfer during subsidiary death. Our model applies to subsidiary closure situations where knowledge transfer occurs within the MNE, i.e., subsidiary activities are to be relocated rather than terminated. Figure 1 depicts the model that resulted from our study.

Our model of knowledge transfer during subsidiary death is guided by previous research on organizational death that emphasizes the process unfolding over time (Crosina & Pratt, 2019; Whetten, 1987). The process of a subsidiary “dying” begins with the announcement of the shut down and culminates with subsidiary closure wherein activities have been relocated. Our model also relates to prior studies of organizational death in that we identify phases that differentiate between discrete stages of the process (Sutton, 1987). In terms of the willingness of subsidiary employees to engage in knowledge transfers, we identify two unique phases of subsidiary death before final closure.

Our model further reveals the centrality and layered nature of subsidiary identity dynamics during the process of knowledge transfer during subsidiary death. Prior research identifies how a subsidiary, such as Gamma, may enjoy a distinct subsidiary identity (Pant & Ramachandran, 2017; Smale, Björkman, Ehrnrooth, John, Mäkelä, & Sumelius, 2015), yet also share the overall MNE meta-identity (Colman et al., 2022; Fortwengel, 2021; Kane, 2010). Sharing the MNE identity allows a subsidiary to be part of the social community of the MNE, enabling knowledge transfers (Kogut & Zander, 1993, 1996). Our model shows how the unexpected announcement of an impending subsidiary closure, a final and disruptive event, renders asunder the subsidiary’s longstanding shared identity with the MNE. This not only caused the absence of a key enabler of MNE knowledge sharing, but also evoked a break in cooperative behavior. Moreover, emotional barriers of subsidiary employees exacerbated this break in cooperative behavior.

Our model then shows how the break in cooperative behavior was addressed through a set of reinforcing subsidiary leadership practices. While the central role of leadership practices in influencing the organizational death process has been observed in a prior study (Sutton, 1987), we detail the specific subsidiary leadership practices influencing the knowledge transfer process within the MNE context. Sensegiving by subsidiary leadership established some sense of continuity in identity at the subsidiary level (see also Corley & Gioia, 2004; Gioia, Schultz, & Corley, 2000). It provided a narrative that connected to the subsidiary’s prior established identity, thereby facilitating the emergence of a legacy subsidiary identity. While this legacy subsidiary identity reflected the change in the subsidiary status during the process of dying (Sutton, 1987), its continuity with the past encouraged employee willingness to engage with knowledge transfers – despite the absence of a shared identity with the MNE.

Moreover, recognizing that the emotional state of individuals influences their response to organizational death, which is a traumatic event (Blau, 2007), our model captures how emotional barriers (including anger and mistrust) trigger a break in cooperative behavior. Further, our model depicts how the emotional responses of employees can be shaped by management (e.g., Vuori, Vuori, & Huy, 2018); in our case, this occurred through subsidiary managers engaging with employee emotions and reconfiguring incentives. Interestingly, these leadership practices acted not only as a traditional extrinsic motivator for employees (in terms of reward), but also reduced emotional barriers (e.g., mistrust, frustration, and discontent). Through these interventions and similar to other cases of organizational death (Sutton, 1987), once emotional barriers were reduced, emotional enablers could take on greater valence as subsidiary death unfolded. This change in the emotions of subsidiary employees helped reinstate cooperative behavior, allowing knowledge transfer to proceed.

Finally, incorporating both individual and organizational factors (including the management of emotions, leadership practices, and the emergence and subsequent leveraging of a legacy subsidiary identity), our model presents a holistic framework for understanding how knowledge transfer can be achieved during subsidiary closure and relocation. It also offers insights into situations where an MNE can no longer rely on a subsidiary sharing its meta-identity or on other traditional ways of motivating employees to share knowledge.

## DISCUSSION

The purpose of this paper is to develop insights into how knowledge is transferred during subsidiary closure and relocations. Based on our findings drawn from a revealing case of subsidiary closure where all activities were relocated within the MNE, we developed a model of knowledge transfer during subsidiary death (Figure 1). This enables three contributions. First, we explore the dynamics of knowledge transfers during subsidiary relocations. Second, we bring novel insights to the growing body of work on subsidiary leadership in an often-overlooked but increasingly important part of subsidiary evolution -  subsidiary closure. Third, we add to the understanding of identity in MNEs. Below, we discuss each of these theoretical implications before detailing boundary conditions, limitations, and future directions for research.

### Knowledge Transfers During Subsidiary Relocations

The international business literature offers few insights into the process of subsidiary relocation, especially as it relates to associated knowledge transfers. The main focus of research into subsidiary divestment has been on understanding why subsidiaries are shut down,  sold or relocated (e.g., Benito, 2005; Berry, 2013; Boddewyn, 1979; Coudounaris, Orero-Blat, & Rodríguez-García, 2020; Ozkan, 2020; Schmid & Morschett, 2020). While prior studies on subsidiary evolution acknowledge that subsidiary closures are a normal aspect of the subsidiary lifecycle (e.g., Birkinshaw, 1996; Birkinshaw & Hood, 1998; Gillmore, 2022; Gillmore, Anderson, & Ekman, 2021; Tippmann et al., 2018), the execution of subsidiary relocations “on the ground” has been underexplored. Addressing this shortcoming, our research introduces the literature on organizational death (e.g., Crosina & Pratt, 2019; Harris & Sutton, 1986; Sutton, 1987) into the domain of international business to understand knowledge transfer during subsidiary relocations.

Kogut and Zander (1993, 1996) establish a shared MNE identity or “longing to belong” within the social community of the MNE as a key mechanism for knowledge transfer across units. We observed that in the aftermath of the subsidiary closure announcement, subsidiary employees no longer share the MNE meta-identity. To our understanding, this is the first study to explore knowledge transfers wherein the condition of a subsidiary sharing an MNE meta-identity (from the perspective of the knowledge sending subsidiary) no longer applies. Importantly, we reveal how alternative mechanisms can promote knowledge transfer in such situations, and when many of the traditional mechanisms for promoting sender willingness and motivation to share knowledge are rendered irrelevant. Our model identifies how subsidiary leadership practices to engage with emotions, reconfigure incentives, and offer sensegiving can create a legacy subsidiary identity that promotes knowledge transfer. We find that these leadership practices can motivate subsidiary employees to continue with familiar practices (such as sharing knowledge with other units) and instill a sense of continued purpose. This adds to our understanding of the range of interventions possible for promoting sender willingness to share knowledge in the absence of a shared MNE identity.

Our findings also spotlight the role of emotions in knowledge transfers. To date, while recognizing the importance of strong emotional ties between MNE organizational units in facilitating knowledge transfer (Nair, Demirbag, Mellahi, & Pillai, 2018), the management of emotions has been a largely overlooked aspect of knowledge sharing within the MNE. However, our findings clearly demonstrate the criticality of effective engagement with employee emotions in knowledge transfer contexts following a disruption or a break in cooperative behavior.

We expect these findings relating to identity and emotions to have implications for knowledge transfer in other situations where there is exclusion from a social community, including aspects of MNE restructuring such as outsourcing and offshoring. In a broader context, these findings could also be relevant to situations where there are retrenchments from alliances, joint ventures, and project partnerships.

### Subsidiary Leadership During Subsidiary Death

Research to date has established the contributions of subsidiary leadership in the growth stages of the subsidiary lifecycle through building subsidiary entrepreneurship (O’Brien, Sharkey Scott, Andersson, Ambos, & Fu, 2019), connecting with the external environment (Cano-Kollmann, Cantwell, Hannigan, Mudambi, & Song, 2016; Lorenzen & Mudambi, 2013), and influencing headquarters (Bouquet, Birkinshaw, & Barsoux, 2016; Conroy et al., 2019; Sarabi, Froese, Chng, & Meyer, 2020). However, further understanding of the activities and contribution of subsidiary leadership activities to the MNE is needed (Meyer, Li, & Schotter, 2020).

By focusing on the activities of subsidiary managers, we detail a set of subsidiary leadership practices – engaging with emotions, sensegiving to enable the emergence of a legacy subsidiary identity, and reconfiguring of incentives – that facilitate the reinstatement of cooperative behavior among subsidiary employees. Importantly, these activities by subsidiary leaders do not simply enable the winding down of subsidiary operations; they also provide for post-death organizing for the MNE through their impact on successfully transferring knowledge from the closing subsidiary. It is also important to note that these practices are of no functional benefit to subsidiary leaders. However, they offer subsidiary leaders an emotional benefit of pride and self-worth.

By revealing leadership activities at the subsidiary level, our findings contrast with prior assumptions that the organizing of subsidiary closure and relocation and associated knowledge transfers fall under the remit of headquarters (Buckley, 2009; Buckley & Strange, 2015). Our analysis shows that, given a loss of trust in headquarters management, the task of ensuring knowledge transfer during subsidiary relocations lies largely in the hands of subsidiary leadership, who have a limited vested interest in the continued success of the MNE. Our study therefore sheds light on a previously hidden consideration for MNEs during subsidiary divestiture. It also substantiates conjectures about the vital role of subsidiary leaders when navigating critical interfaces between headquarters and subsidiary employees (Schotter & Beamish, 2011; Schotter, Mudambi, Doz, & Gaur, 2017) and when managing sensitive relationships on behalf of the MNE (Meyer et al., 2020).

### Identity within MNEs

Our findings further contribute to theories on identity in MNEs (e.g., Fortwengel, 2021; Pant & Ramachandran, 2017). First, we complement existing theory in showing the benefits of some continuity in subsidiary identity following a disruptive event. Work on organizational spinoffs (Corley & Gioia, 2004) and mergers (Clark, Gioia, Ketchen, & Thomas, 2010; Maguire & Phillips, 2008) has established that identity often changes over time (Eury et al., 2018; Gioia et al., 2013; Pant & Ramachandran, 2017) or in response to key events – particularly following a disruption (Clark et al., 2010). While the impact on identity in such circumstances offers leaders an opportunity to reconstruct new understandings of “who they are” (Corley & Gioia, 2004), our study shows that it may be desirable to offer a strong anchor in past subsidiary identity through the creation of a legacy subsidiary identity. Such an identity can help maintain the established routines, practices, and behavioral norms that are of continued relevance to the activities and task obligations of the subsidiary and MNE. Building upon Gioia et al. (2000), we highlight the fluid nature of identity, differentiating between an “enduring” identity and the notion of an identity as having “continuity.” While an enduring identity implies that it has permanency and remains the same over time, identity as continuous captures that it is malleable and capable of retaining certain beliefs and values over time and context (Gioia et al., 2000: 65). The emergence and subsequent leveraging of a legacy identity in this study demonstrates how identity continuity can be married with responsiveness, as subsidiary identity can evolve both over time and in response to significant disruption.

Second, our findings have implications for the relationship between subsidiary identity and a shared MNE meta-identity. While distinct, a subsidiary identity is usually considered as nested within the meta-identity of the MNE (Fortwengel, 2021; Pant & Ramachandran, 2017). In contrast, our findings show that a legacy subsidiary identity may no longer be embedded within a MNE meta-identity. However, as the legacy subsidiary identity, at least in the case of Gamma, encapsulated behavioral norms that were desirable for the MNE, this disconnect did not negatively impact the functioning of the MNE. Still, this finding may have implications for other contexts where a legacy subsidiary identity may not be so favorable – particularly in the long-standing challenge of achieving successful knowledge transfer following subsidiary acquisitions (Bresman, Birkinshaw, & Nobel, 2010; Sarala & Vaara, 2010; Zhou, Fey, & Yildiz, 2020).

### Boundary Conditions

While based on the study of the closure of an established subsidiary of a U.S. MNE in Europe where all activities needed to be relocated, we believe many aspects of our model have broader relevance. It is plausible that our findings hold in other situations of relocating activities in global value chains, especially in situations where tacit and organizationally complex knowledge is involved and job loss is inevitable (e.g., the termination of partnerships, alliances, and joint ventures). Such situations are likely to evoke emotional barriers and quash motivation in the affected unit, leading to disengagement from knowledge transfer activities.

In our study, the subsidiary had a well-established identity and the closure decision represented a noticeable disruption to this long-standing identity. It is likely that in situations where subsidiary employees are primed for the closure of their site, emotional barriers can be dampened. However, we nonetheless expect the thrust of our model to apply in that it would still be necessary to re-instill the motivation that would enable the effective relocation of subsidiary activities. Similarly, we expect our model to remain relevant in situations where the subsidiary is located in jurisdictions with weaker statutory rights and less generous norms for supporting employees affected by compulsory redundancy. In such situations, our finding of the need to create a legacy subsidiary identity and engage subsidiary leadership practices may be very relevant to motivate subsidiary employees before they move to a new job.

### Limitations and Future Research

The boundary conditions detailed above provide opportunities for future research. We note, too, that our study unfolds mainly from the perspective of the subsidiary. This aligns with our intention to build theory for this under-explored phenomenon, which requires being close to key informants. We did conduct interviews with an informant who relocated to HQ and therefore had insights from the perspective of the sites that received knowledge. Nevertheless, a fuller perspective of headquarters and knowledge receivers would allow for further corroboration and refinement of the model. Similarly, as our data on employee emotions is primarily based on managers’ perception, capturing data from all layers of the organization would have further corroborated our findings. In any case, it seems promising for future research to pay more attention to emotions in knowledge transfers, headquarters–subsidiary relationships, and in situations where subsidiary employees are surprised by the decisions of headquarters as their affective experiences may explain organizational outcomes.

### Managerial Implications

As far as we are aware, this study is the first to provide valuable insights into the management of knowledge transfer during subsidiary closure or death. Our insights therefore start to fill a void and can guide management across the MNE if subsidiary closure and relocation situations arise.

Our model of knowledge transfer during subsidiary death can help subsidiary leadership anticipate challenges at critical junctions in the relocation process. At the same time, it also reveals leadership practices that can help in dealing with those challenges. Specifically, these insights allow subsidiary leaders to anticipate employee reaction to closure, identify the mechanisms needed to support the emotional responses of employees, and deal with a break in subsidiary identity. Further, the findings provide guidance on the leadership practices that support the reinstatement of cooperative behavior among subsidiary employees, thereby enabling knowledge transfer.

The case of Gamma vividly illustrates how high performance, competence development, a history of initiative generation, and a strong headquarters–subsidiary relationship may fail to protect a subsidiary from a closure decision. This cautionary tale should prompt subsidiary leaders to constantly consider the position of their subsidiary, as headquarters makes decisions from an organizational perspective. Subsidiary and HQ leaders should also carefully consider how and when they communicate an impending closure. In the Gamma case, the unexpected nature of the announcement led to surprise and emotional unpreparedness among subsidiary employees. Emotional barriers then heightened the challenges of knowledge transfer.

From a headquarters perspective, our study provides rich insights into the dynamics of subsidiary closure, the potential to underestimate the value of the knowledge held by the subsidiary, and the difficulties of motivating subsidiary employees to transfer business-critical knowledge in closure situations.

## CONCLUSION

In an increasingly challenging global context, subsidiary closure and relocations are a normal and growing element of MNE management. Our longitudinal case study of a subsidiary as it experienced the emotionally charged process of closure and relocation of activities provides a revelatory account of how subsidiary knowledge can be transferred when subsidiary employees no longer share the MNE meta-identity. Offering rich new theoretical insights, our model identifies the role of emotions as barriers and enablers, and shows how leadership practices are critical to the emergence of a legacy subsidiary identity for the transfer of knowledge in such situations.

## NOTES

1. Note that in this study and the process model we advance, we use the terms “subsidiary relocation” and “subsidiary death” interchangeably. This is informed by the contextual circumstances of the study, whereby a subsidiary will cease to exist and all of its activities will be relocated to other sites within the MNE.
2. It is important to note that subsidiary leaders, as subsidiary employees, may share the collective emotional response by subsidiary employees. However, given their leadership role, their actions may also influence the process of subsidiary death.

## Appendix: Interview Guides

### Sample Questions from Interviews during Subsidiary Relocation

- Please tell me about your current or previous roles.
- What was the role of the subsidiary within the MNE?
- Where have/will activities been moved to?
- Since the initial announcement of the closure, what supports (if any) have been put in place by headquarters and subsidiary management to facilitate the smooth closure of the subsidiary?
- How did the receiving site(s) prepare?
- If applicable, how have subsidiary employees been involved in the transfer of existing knowledge to other sites?
- If applicable, how are these transfers managed?
- What challenges (if any) did the closing of the subsidiary create for management locally? How do you deal with them?
- What challenges (if any) did the closing of the subsidiary create for headquarters management? How do they deal with them?
- Is there anything that you would like to add?

### Sample Questions from Follow-up Interviews

- When the closure was announced, can you please describe the reaction of staff?
- How did staff initially feel about the closure?
- How would you describe the role of local management and headquarters management in dealing with those reactions/feelings?
- How would you describe how those reactions/feelings changed over time?
- Where changes were observed, what do you think caused this change?
- What was the role of local and headquarters management in influencing this change?
- Towards the end, how did the staff feel?
- What factors assisted in transferring knowledge or activities to other sites?
- What risks were there to business if knowledge could not be transferred or if substantial knowledge was lost?

## References

[CR138] Albert, S., & Whetten, D. A. 1985. Organizational identity. *Research in Organizational Behavior*, 7: 263–295.

[CR1] Albert S, Ashforth BE, Dutton JE (2000). Organizational identity and identification: Charting new waters and building new bridges. Academy of Management Review.

[CR2] Ashforth BE, Rogers KM, Corley KG (2011). Identity in organizations: Exploring cross-level dynamics. Organization Science.

[CR3] Ashforth BE, Schinoff BS, Brickson SL (2020). My company is friendly”, “Mine's a Rebel”: Anthropomorphism and shifting organizational identity from “What” to “Who. Academy of Management Review.

[CR4] Athanassiou N, Nigh D (2000). Internationalization, tacit knowledge, and the top management teams of MNCs. Journal of International Business Studies.

[CR5] Bakker M, Leenders RTAJ, Gabbay SM, Kratzer J, Engelen V, Jo ML (2006). Is trust really social capital? Knowledge sharing in product development projects. The Learning Organization.

[CR6] Baldwin A, McClelland D, Baldwin A, Bronfenbrenner U, Strodtbeck F (1959). The role of an "ability" construct in a theory of behavior. Talent and society.

[CR7] Balogun J, Fahy K, Vaara E (2019). The interplay between HQ legitimation and subsidiary legitimacy judgments in HQ relocation: A social psychological approach. Journal of International Business Studies.

[CR8] Balogun J, Jarzabkowski P, Vaara E (2011). Selling, resistance and reconciliation: A critical discursive approach to subsidiary role evolution in MNEs. Journal of International Business Studies.

[CR9] Belderbos R, Zou J (2006). Foreign investment, divestment and relocation by Japanese electronics firms in East Asia. Asian Economic Journal.

[CR10] Belderbos R, Zou J (2009). Real options and foreign affiliate divestments: A portfolio perspective. Journal of International Business Studies.

[CR11] Bell E (2012). Ways of seeing organisational death: A critical semiotic analysis of organisational memorialization. Visual Studies.

[CR12] Bell E, Taylor S (2011). Beyond letting go and moving on: New perspectives on organizational death, loss and grief. Scandinavian Journal of Management.

[CR13] Benito GRG (2005). Divestment and international business strategy. Journal of Economic Geography.

[CR14] Berends H, Deken F (2021). Composing qualitative process research. Strategic Organization.

[CR15] Berry H (2010). Why do firms divest?. Organization Science.

[CR16] Berry H (2013). When do firms divest foreign operations?. Organization Science.

[CR17] Birkinshaw J (1996). How multinational subsidiary mandates are gained and lost. Journal of International Business Studies.

[CR18] Birkinshaw J, Hood N (1998). Multinational subsidiary evolution: Capability and charter change in foreign-owned subsidiary companies. Academy of Management Review.

[CR19] Blau G (2006). A process model for understanding victim responses to worksite/function closure. Human Resource Management Review.

[CR20] Blau G (2007). Partially testing a process model for understanding victim responses to an anticipated worksite closure. Journal of Vocational Behavior.

[CR21] Blau G (2008). Exploring antecedents of individual grieving stages during an anticipated worksite closure. Journal of Occupational and Organizational Psychology.

[CR22] Boddewyn J (1979). Foreign divestment: Magnitude and factors. Journal of International Business Studies.

[CR23] Bouquet C, Birkinshaw J, Barsoux JL (2016). Fighting the headquarters knows best syndrome. Sloan Management Review.

[CR24] Bresman H, Birkinshaw J, Nobel R (2010). Knowledge transfer in international acquisitions. Journal of International Business Studies.

[CR25] Buckley PJ (2009). The impact of the global factory on economic development. Journal of World Business.

[CR26] Buckley PJ, Strange R (2015). The governance of the global factory: Location and control of world economic activity. Academy of Management Perspectives.

[CR27] Cabrera A, Cabrera EF (2002). Knowledge-sharing dilemmas. Organization Studies.

[CR28] Calder BJ, Staw BM (1975). The self-perception of intrinsic and extrinsic motivation. Journal of Personality and Social Psychology.

[CR29] Cano-Kollmann M, Cantwell J, Hannigan TJ, Mudambi R, Song J (2016). Knowledge connectivity: An agenda for innovation research in international business. Journal of International Business Studies.

[CR30] Cantwell J, Mudambi R (2005). MNE competence-creating subsidiary mandates. Strategic Management Journal.

[CR31] Chang HH, Chuang S-S (2011). Social capital and individual motivations on knowledge sharing: Participant involvement as a moderator. Information and Management.

[CR32] Clark E, Geppert M (2011). Subsidiary integration as identity construction and institution building: A political sensemaking approach. Journal of Management Studies.

[CR33] Clark SM, Gioia DA, Ketchen DJ, Thomas JB (2010). Transitional identity as a facilitator of organizational identity change during a merger. Administrative Science Quarterly.

[CR34] Colman HL, Grøgaard B, Stensaker IG (2022). Organizational identity work in MNE subsidiaries: Managing dual embeddedness. Journal of International Business Studies.

[CR35] Conroy KM, Collings DG, Clancy J (2019). Sowing the seeds of subsidiary influence: Social navigating and political maneuvering of subsidiary actors. Global Strategy Journal.

[CR36] Corbin J, Strauss A, Corbin J, Strauss A (2008). Strategies for qualitative data analysis. Basics of qualitative research. Techniques and procedures for developing grounded theory.

[CR37] Corley KG, Gioia DA (2004). Identity ambiguity and change in the wake of a corporate spin-off. Administrative Science Quarterly.

[CR38] Cornelissen J (2017). Editor’s comments: Developing propositions, a process model, or a typology? Addressing the challenges of writing theory without a boilerplate. Academy of Management Review.

[CR39] Coudounaris DN, Orero-Blat M, Rodríguez-García M (2020). Three decades of subsidiary exits: Parent firm financial performance and moderators. Journal of Business Research.

[CR40] Crosina E, Pratt MG (2019). Toward a model of organizational mourning: The case of former Lehman Brothers bankers. Academy of Management Journal.

[CR41] Cruz NM, Perez VM, Cantero CT (2009). The influence of employee motivation on knowledge transfer. Journal of Knowledge Management.

[CR42] Cuervo-Cazurra A, Andersson U, Brannen MY, Nielsen BB, Reuber AR (2016). From the editors: Can I trust your findings? Ruling out alternative explanations in international business research. Journal of International Business Studies.

[CR43] Cunningham J (1997). Feelings and interpretations during an organization’s death. Journal of Organizational Change Management.

[CR44] Davenport TH, Prusak L (1998). Working knowledge.

[CR45] Dörrenbächer C, Gammelgaard J (2011). Subsidiary power in multinational corporations: The subtle role of micro-political bargaining power. Critical Perspectives on International Business.

[CR46] Doz Y, Santos J, Williamson P (2001). From global to metanational: How companies win in the knowledge economy.

[CR47] Edman J (2016). Cultivating foreignness: How organizations maintain and leverage minority identities. Journal of Management Studies.

[CR48] Erkama N, Vaara E (2010). Struggles over legitimacy in global organizational restructuring: A rhetorical perspective on legitimation strategies and dynamics in a shutdown case. Organization Studies.

[CR49] Eury JL, Kreiner GE, Treviño LK, Gioia DA (2018). The past is not dead: Legacy identification and alumni ambivalence in the wake of the Sandusky scandal at Penn State. Academy of Management Journal.

[CR50] Flick U (1992). Triangulation revisited: Strategy of validation or alternative?. Journal for the Theory of Social Behaviour.

[CR51] Fortwengel J (2021). The formation of an MNE identity over the course of internationalization. Journal of International Business Studies.

[CR52] Foss NJ, Minbaeva DB, Pedersen T, Reinholt M (2009). Encouraging knowledge sharing among employees: How job design matters. Human Resource Management.

[CR53] Friesl M, Silberzahn R (2017). Managerial coordination challenges in the alignment of capabilities and new subsidiary charters in MNEs. Organization Studies.

[CR54] Gaines R (1997). Detachment and continuity: The two tasks of mourning. Contemporary Psychoanalysis.

[CR55] Gao Y, Riley M (2010). Knowledge and identity: A review. International Journal of Management Reviews.

[CR56] Gaur AS, Ma H, Ge B (2019). MNC strategy, knowledge transfer context, and knowledge flow in MNCs. Journal of Knowledge Management.

[CR57] George A, Bennett A, Lynn-Jones S, Miller S (2005). Case studies and theory development in the social sciences.

[CR58] Gillmore E (2022). Mandate dynamics and the importance of mandate loss for subsidiary evolution. International Business Review.

[CR59] Gillmore E, Andersson U, Ekman P (2021). The enduring effects of relational attributes on subsidiary evolution after mandate loss. Global Strategy Journal.

[CR60] Gioia DA, Chittipeddi K (1991). Sensemaking and sensegiving in strategic change initiation. Strategic Management Journal.

[CR61] Gioia DA, Patvardhan SD, Hamilton AL, Corley KG (2013). Organizational identity formation and change. Academy of Management Annals.

[CR62] Gioia DA, Schultz M, Corley KG (2000). Identity, image, and adaptive instability. Academy of Management Review.

[CR63] Grodal S, Anteby M, Holm A (2021). Achieving rigor in qualitative analysis: The role of active categorization in theory building. Academy of Management Review.

[CR64] Gupta AK, Govindarajan V (1991). Knowledge flows and the structure of control within multi-national corporations. Academy of Management Review.

[CR65] Gupta AK, Govindarajan V (2000). Knowledge flows within multinational corporations. Strategic Management Journal.

[CR66] Harris SG, Sutton RI (1986). Functions of parting ceremonies in dying organizations. Academy of Management Journal.

[CR67] Hartwell CA, Devinney T (2021). Populism, political risk, and pandemics: The challenges of political leadership for business in a post-COVID world. Journal of World Business.

[CR68] Husted K, Michailova S, Minbaeva DB, Pedersen T (2012). Knowledge-sharing hostility and governance mechanisms: An empirical test. Journal of Knowledge Management.

[CR69] Kane AA (2010). Unlocking knowledge transfer potential: Knowledge demonstrability and superordinate social identity. Organization Science.

[CR70] Kano L, Tsang EWK, Yeung HW-C (2020). Global value chains: A review of the multi-disciplinary literature. Journal of International Business Studies.

[CR71] Kogut B, Zander U (1993). Knowledge of the firm and the evolutionary theory of the multinational corporation. Journal of International Business Studies.

[CR72] Kogut B, Zander U (1996). What firms do? Coordination, identity and learning. Organization Science.

[CR73] Konara P, Ganotakis P (2020). Firm-specific resources and foreign divestments via selloffs: Value is in the eye of the beholder. Journal of Business Research.

[CR75] Kouamé S, Liu F (2020). Capturing emotions in qualitative strategic organization research. Strategic Organization.

[CR76] Kreiner GE, Ashforth BE (2004). Evidence toward an expanded model of organizational identification. Journal of Organizational Behavior.

[CR77] Kreiner GE, Hollensbe E, Sheep ML, Smith BR, Kataria N (2015). Elasticity and the dialectic tensions of organizational identity: How can we hold together while we’re pulling apart?. Academy of Management Journal.

[CR78] Langley A (1999). Strategies for theorizing from process data. Academy of Management Review.

[CR79] Lee TW (1999). Using qualitative methods in organizational research.

[CR80] Lin HF (2007). Effects of extrinsic and intrinsic motivation on employee knowledge sharing intentions. Journal of Information Science.

[CR81] Lincoln YS, Guba EG (1985). Naturalistic inquiry.

[CR137] Liu Yipeng, Meyer Klaus E. (2020). Boundary spanners, HRM practices, and reverse knowledge transfer: The case of Chinese cross-border acquisitions. Journal of World Business.

[CR82] Lorenzen M, Mudambi R (2013). Clusters, connectivity and catch-up: Bollywood and Bangalore in the global economy. Journal of Economic Geography.

[CR83] Maguire S, Phillips N (2008). ‘‘Citibankers’’ at Citigroup: A study of the loss of institutional trust after a merger. Journal of Management Studies.

[CR84] Maitlis S, Christianson M (2014). Sensemaking in organizations: Taking stock and moving forward. Academy of Management Annals.

[CR85] Maitlis S, Lawrence TB (2007). Triggers and enablers of sensegiving in organizations. Academy of Management Journal.

[CR86] Maitlis S, Vogus TJ, Lawrence TB (2013). Sensemaking and emotion in organizations. Organizational Psychology Review.

[CR87] Mantere S, Ketokivi M (2013). Reasoning in organization science. Academy of Management Review.

[CR88] Mees-Buss J, Welch C, Westney E (2019). What happened to the transnational? The emergence of the neo-global corporation. Journal of International Business Studies.

[CR89] Meyer KE, Li C, Schotter AP (2020). Managing the MNE subsidiary: Advancing a multi-level and dynamic research agenda. Journal of International Business Studies.

[CR90] Minbaeva D (2007). Knowledge transfer in multinational corporations. Management International Review.

[CR91] Minbaeva D, Michailova S (2004). Knowledge transfer and expatriation in multinational corporations: The role of disseminative capacity. Employee Relations.

[CR92] Minbaeva D, Pederson T, Bjorkman I, Fey C, Park H (2003). MNC knowledge transfer, subsidiary absorptive capacity and HRM. Journal of International Business Studies.

[CR93] Monteiro FL, Arvidsson M, Birkinshaw J (2008). Knowledge flows within multinational corporations: explaining subsidiary isolation and its performance implications. Organization Science.

[CR94] Mudambi R, Piscitello L, Rabbiosi L (2014). Reverse knowledge transfer in MNEs: Subsidiary innovativeness and entry modes. Long Range Planning.

[CR95] Nachum L, Song S (2011). The MNE as a portfolio: Interdependencies in MNE growth trajectory. Journal of International Business Studies.

[CR96] Nair SR, Demirbag M, Mellahi K, Pillai KG (2018). Do parent units benefit from reverse knowledge transfer?. British Journal of Management.

[CR97] Nonaka I (1994). A dynamic theory of organizational knowledge creation. Organization Science.

[CR98] Nonaka I, Takeuchi H (1995). The knowledge creating company.

[CR99] Noorderhaven N, Harzing AW (2009). Knowledge-sharing and social interaction within MNEs. Journal of International Business Studies.

[CR100] O’Brien D, Scott PS, Andersson U, Ambos TC, Fu N (2019). The microfoundations of subsidiary initiatives: How subsidiary manager activities unlock entrepreneurship. Global Strategy Journal.

[CR101] Osterloh M, Frey BS (2000). Motivation, knowledge transfer, and organizational forms. Organization Science.

[CR140] Ozkan Kubilay S.L. (2020). International market exit by firms: Misalignment of strategy with the foreign market risk environment. International Business Review.

[CR102] Pant A, Ramachandran J (2017). Navigating identity duality in multinational subsidiaries: A paradox lens on identity claims at Hindustan Unilever 1959–2015. Journal of International Business Studies.

[CR103] Parker A, Tippmann E, Kratochvil R (2019). Accessing diverse knowledge for problem solving in the MNC: A network mobilization perspective. Global Strategy Journal.

[CR104] Pettigrew AM (1990). Longitudinal field research on change: Theory and practice. Organization Science.

[CR105] Polanyi M (1966). The tacit dimension.

[CR106] Reinholt M, Pedersen T, Foss NJ (2011). Why a central network position isn’t enough: The role of motivation and ability for knowledge sharing in employee networks. Academy of Management Journal.

[CR139] Pratt, M. G., & Kraatz, M. S. 2009. E pluribus unum: Multiple identities and the organizational self. In L. M. Roberts & J. E. Dutton (Eds.), *Exploring positive identities and organizations: Building a theoretical and research foundation*: 385–410. New York: Psychology Press.

[CR107] Reuber AR, Fischer E (2021). Putting qualitative international business research in context(s). Journal of International Business Studies..

[CR108] Rugman AM, Verbeke A (2001). Subsidiary-specific advantages in multinational enterprises. Strategic Management Journal.

[CR109] Sarabi A, Froese F, Chng DHM, Meyer KE (2020). Entrepreneurial leadership and MNE subsidiary performance: The moderating role of subsidiary context. International Business Review.

[CR110] Sarala RM, Vaara E (2010). Cultural differences, convergence, and crossvergence as explanations of knowledge transfer in international acquisitions. Journal of International Business Studies.

[CR111] Schmid D, Morschett D (2020). Decades of research on foreign subsidiary divestment: What do we really know about its antecedents?. International Business Review..

[CR112] Schotter A, Beamish PW (2011). Performance effects of MNC headquarters–subsidiary conflict and the role of boundary spanners: The case of headquarter initiative rejection. Journal of International Management.

[CR113] Schotter A, Mudambi R, Doz YL, Gaur A (2017). Boundary spanning in global organizations. Journal of Management Studies.

[CR114] Smale A, Björkman I, Ehrnrooth M, John S, Mäkelä K, Sumelius J (2015). Dual values-based organizational identification in MNC subsidiaries: A multilevel study. Journal of International Business Studies.

[CR115] Song S (2014). Subsidiary divestment: The role of multinational flexibility. Management International Review.

[CR116] Sonnenfeld, J., et al. 2022. Yale School of Management. Almost 1,000 companies have curtailed operations in Russia—bur some remain. https://som.yale.edu/story/2022/almost-1000-companies-have-curtailed-operations-russia-some-remain. Accessed 9 June 2022.

[CR117] Stendahl E, Schriber S, Tippmann E (2021). Control changes in multinational corporations: Adjusting control approaches in practice. Journal of International Business Studies..

[CR118] Sutton RI (1987). The process of organizational death: Disbanding and reconnecting. Administrative Science Quarterly.

[CR119] Szulanski G (1996). Exploring internal stickiness: Impediments to the transfer of best practice within the firm. Strategic Management Journal.

[CR120] Tallman S, Chacar AS (2011). Knowledge accumulation and dissemination in MNEs: A practice-based framework. Journal of Management Studies.

[CR121] Thomas DC, Cuervo-Cazurra A, Brannen MY (2011). Explaining theoretical relationships in international business research: It’s about the arrows, NOT the boxes. Journal of International Business Studies.

[CR122] Tippmann E, Sharkey Scott P, Mangematin V (2012). Problem solving in MNCs: How local and global solutions are (and are not) created. Journal of International Business Studies.

[CR123] Tippmann E, Sharkey Scott P, Reilly M, O’Brien D (2018). Subsidiary coopetition competence: Navigating subsidiary evolution in the multinational corporation. Journal of World Business.

[CR124] UNCTAD (2020). World investment report: International production beyond the pandemic.

[CR125] Vaara E, Tienari J (2011). On the narrative construction of multinational corporations: An antenarrative analysis of legitimation and resistance in a cross-border merger. Organization Science.

[CR126] Van de Ven AH, Poole MS (1995). Explaining development and change in organizations. Academy of Management Review.

[CR127] Voisey CJ (2010). When a Japanese subsidiary is not a Japanese subsidiary: Internationalization as changing organizational identity and capabilities. International Journal of Cross Cultural Management.

[CR128] Vuori N, Vuori TO, Huy QN (2018). Emotional practices: how masking negative emotions impacts the post-acquisition integration process. Strategic Management Journal.

[CR129] Vuori TO, Huy QN (2016). Distributed attention and shared emotions in the innovation process: How Nokia lost the smartphone battle. Administrative Science Quarterly.

[CR130] Walsh IJ, Bartunek JM (2011). Cheating the fates: Organizational foundings in the wake of demise. Academy of Management Journal.

[CR131] Walsh IJ, Bartunek JM (2012). Rising from the ashes: Appreciating and fostering post-death organizing. Organizational Dynamics.

[CR132] Whetten DA (1987). Organizational growth and decline processes. Annual Review of Sociology.

[CR133] Wu Y, Strange R, Shirodkar V (2021). MNE divestments of foreign affiliates: Does the strategic role of the affiliate have an impact?. Journal of Business Research.

[CR134] Yin R (2014). Case study research: Design and methods.

[CR135] Zeng R, Grøgaard B, Steel P (2018). Complements or substitutes? A Meta-analysis of the role of integration mechanisms for knowledge transfer in the MNE network. Journal of World Business.

[CR136] Zhou AJ, Fey C, Yildiz HE (2020). Fostering integration through HRM practices: An empirical examination of absorptive capacity and knowledge transfer in cross-border M&As. Journal of World Business.

